# Recent Advances in the Development of Sigma Receptor Ligands as Cytotoxic Agents: A Medicinal Chemistry Perspective

**DOI:** 10.1021/acs.jmedchem.0c02265

**Published:** 2021-06-02

**Authors:** Antonino N. Fallica, Valeria Pittalà, Maria N. Modica, Loredana Salerno, Giuseppe Romeo, Agostino Marrazzo, Mohamed A. Helal, Sebastiano Intagliata

**Affiliations:** † Department of Drug and Health Sciences, University of Catania, Viale A. Doria 6, 95125 Catania, Italy; ‡ University of Science and Technology, Biomedical Sciences Program, Zewail City of Science and Technology, October Gardens, sixth of October, Giza 12578, Egypt; § Medicinal Chemistry Department, Faculty of Pharmacy, Suez Canal University, Ismailia 41522, Egypt

## Abstract

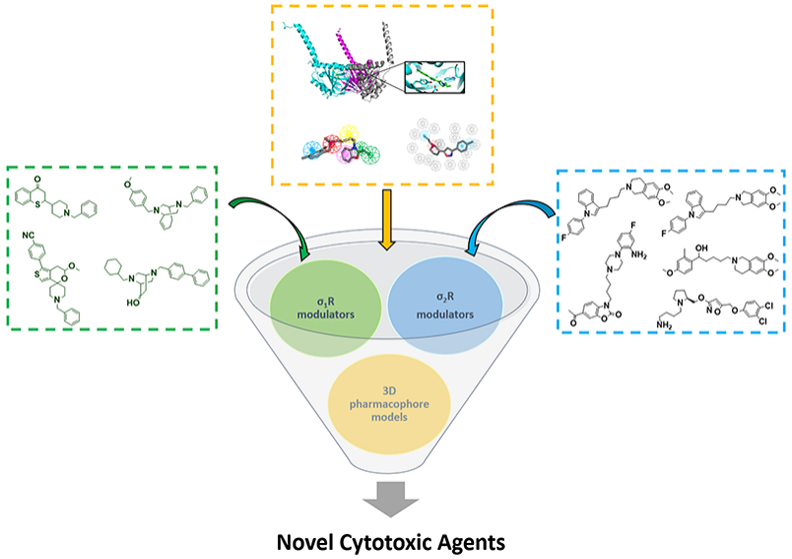

Since their discovery as distinct receptor proteins, the specific physiopathological role of sigma receptors (σRs) has been deeply investigated. It has been reported that these proteins, classified into two subtypes indicated as σ_1_ and σ_2_, might play a pivotal role in cancer growth, cell proliferation, and tumor aggressiveness. As a result, the development of selective σR ligands with potential antitumor properties attracted significant attention as an emerging theme in cancer research. This perspective deals with the recent advances of σR ligands as novel cytotoxic agents, covering articles published between 2010 and 2020. An up-to-date description of the medicinal chemistry of selective σ_1_R and σ_2_R ligands with antiproliferative and cytotoxic activities has been provided, including major pharmacophore models and comprehensive structure–activity relationships for each main class of σR ligands.

## 1 Introduction

Cancer is a severe health concern, and it is the second leading cause of death globally.^1^ According to a World Health Organization report (2020), the global cancer burden is significant and increasing, with an estimated 9.6 million deaths worldwide from cancer in 2018. About 300,000 new cases per year have been diagnosed among children aged 0–19 years, while the calculated total annual cost of cancer in 2010 was more than US $1 trillion worldwide. Usually, treatment options include chemotherapy, radiotherapy, and surgery. Frequently, the toxicity of large doses of chemotherapy and the lack of effectiveness in certain tumors make surgery and radiotherapy the preferred options. By definition, anticancer drugs target rapidly multiplying cells, leading to variable toxicities to the gastrointestinal tract, bone marrow, hair follicles, and gonads. Hematological toxicity manifests as acute cytopenia due to the cytotoxic effect on the hematopoietic precursor cells. The gastrointestinal toxicity, nausea, vomiting, and anorexia is usually a physiological reflex to remove toxic substances from the gastrointestinal tract. Nausea is a widespread side effect among chemotherapeutic agents and entails 5-HT_3_ antagonists such as ondansetron in several situations. Another common toxicity is hair follicle damage which represents both physiological and psychological burdens for the patients. Alopecia usually develops due to the cytotoxic effect of the drug on the rapidly dividing hair follicles. Finally, neurotoxicity can occur with drugs that cross the blood–brain barrier, such as vincristine, 5-fluorouracil, thiotepa, and cisplatin. Some other agents can cause peripheral neuropathy, such as paclitaxel and carboplatin. Besides, several factors may affect the effectiveness of the chemotherapy regimen, including early intracellular drug inactivation, overexpression of drug efflux pumps, low drug uptake, or dysregulation of specific intracellular signaling pathways targeted by the therapeutic drugs. Despite the striking results obtained by tumor immunotherapy and nanomedicine,^2,3^ several issues still need to be overcome. These factors emphasize the need to identify and validate alternative biological targets mainly detectable in tumor cells and develop novel anticancer agents with enhanced efficacy, safety profile, and compliance.^4,5^

Sigma receptors (σRs) are considered promising targets for treating different heterogeneous medical conditions, including cancer.^6−8^ The history of σRs began in the early 1970s when Martin et al. proposed the involvement of a subtype of the opioid receptor family, named the “σ-opioid” receptors, in the psychotomimetic effects caused by the putative opioid agonist *N*-allylnormetazocine derivative (±)-SKF-10,047 in the spinal dog model.^9,10^ However, later on, the binding site within this protein, whose gene sequence was cloned by Su and colleagues,^11^ was unresponsive to naloxone and naltrexone, suggesting the distinction of this type of protein from opioid receptors. In the following years, SKF-10,047 was found to interact with many biological targets, adding confusion about the classification of σRs.^12,13^ Further studies on this compound determined that it could not be completely displaced from its receptor using selective opioid ligands, indicating that it was bound to another distinct receptor. More specifically, racemic SKF-10,047 has the ability to produce algesia and psychotomimetic effects in humans. The analgesic effect is believed to be mediated through the action of (−)-SKF-10,047 on the μ and *k* opioid receptors. Conversely, it was found that (+)-SKF-10,047 binds with very low affinity to both opioid receptors, and its pharmacological action is mediated through a different site.^14^ This other site has since been designated as the σR.^15^ Further studies proved that σRs are a non-opioid, non-GPCR transmembrane protein expressed mainly in the endoplasmic reticulum (ER) membrane and physically associated with the mitochondria.^16^ σRs act as chaperone proteins that interfere with ion-channels and GPCR receptors activity modulating several physiological pathways through ER stress and control of intracellular Ca^2+^ homeostasis.^17^ Ultimately, σRs have been classified into two subtypes, σ_1_ and σ_2_ receptors, depending on their biological actions, distribution, sizes, and other factors.^8^

**Figure 1:**
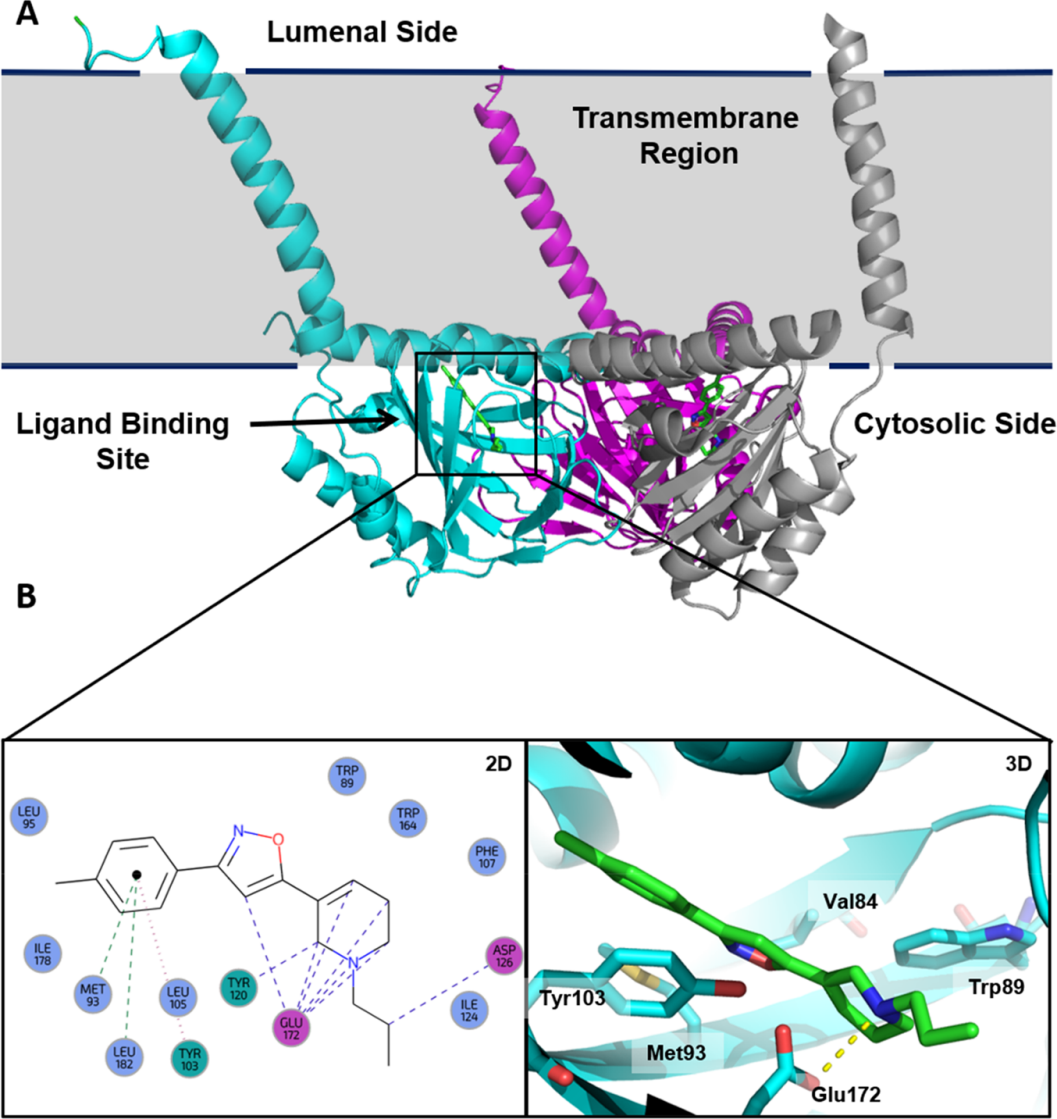
(A) Cartoon representation of the σ_1_R crystal structure (PDB ID: 5HK1); each color outlines a distinguished σ_1_R protomer which forms the σ_1_R trimer. (B) 2D and 3D representation of protein–ligand interactions of the σ_1_R with PD144418 (PDB ID: 5HK1). The ionic bond between the basic nitrogen of PD144418 and the Glu172 amino acid residue is shown as a dashed yellow line.

It was only in 1996 that the gene sequence of σ_1_R has been cloned by Hanner et al.^18^ and was found to be expressed in various tissues inside and outside the CNS. The σ_1_R subtype has been cloned from many species, including mice, rats, guinea pigs, and humans. It is a 223 amino acid protein with a molecular weight of about 24 kDa. σ_1_Rs are widely distributed in several tissues, and they are present in the brain, spinal cord, and peripheral nerves.^19^ A breakthrough occurred in 2016 with the publication of the three-dimensional (3D) crystal structure of the human σ_1_R by Schmidt and co-workers.^20^ The reported structures showed a trimeric architecture formed by the association of three identical protomers possessing a single transmembrane domain (Figure 1A).^20^ So far, five different crystal structures of the σ_1_R in complex with historical σ_1_R ligands (i.e., (+)-pentazocine, haloperidol, NE-100, PD144418, and 4-IBP) have been reported,^20,21^ which have revealed a preserved ligand’s binding mode with high similarity shared by different chemical classes. Notably, the ligand-binding site is deeply located inside the large β-barrel region where ligands are accommodated in a very hydrophobic pocket entirely occluded from solvent molecules.

Analysis of the protein–ligand contacts outlines a major ionic bond involving the basic nitrogen of the σ_1_R ligand (e.g., PD144418, Figure 1B) and the Glu172 amino acid residue as well as multiple hydrophobic interactions with bulky hydrophobic residues (i.e., Val84, Trp89, Met93, Tyr103) that shape the internal edge of the binding pocket.

Growing evidence implicates the σ_1_R in various neurological disorders such as depression, anxiety, schizophrenia, and Alzheimer’s disease.^22^ Recent studies suggest that σ_1_R modulators possess the therapeutic potential to treat drug abuse.^23^ Interestingly, recent studies have investigated the repurposing of σ_1_R ligands for interfering with the early stages of severe acute respiratory syndrome coronavirus 2 (SARS-CoV-2) replication. This approach was inspired by the colocalization of σ_1_R with the viral replicase protein in the ER membrane and its interaction with the nonstructural SARS-CoV-2 protein Nsp6.^24,25^ Nowadays, the oncogenic role of σ_1_Rs has not been fully elucidated. It is known that this protein is overexpressed in a wide number of cancer cell lines and σ_1_Rs fully functional activity is required for proper growth, proliferation, migration, and survival of cancer cells. The use of σ_1_R negative modulators, often considered as “σ_1_R antagonists”, or σ_1_ gene silencing through the application of RNAi hamper tumor cell growth and survival.^6^ Contrarily, the overexpression of σ_1_Rs through recombinant techniques or the σ_1_Rs positive modulation exerted by selective small molecules, often considered as “σ_1_R agonists”, causes opposite effects.^6^

Unlike the σ_1_R, up to now, the crystal structure of the σ_2_R is still unknown. The difficulty of its isolation and purification is mainly due to its distribution in the lipid environment and its low abundance in the prepared mammalian membranes.^12^ The genetic identity of the σ_2_R was revealed recently in 2017 when Alon et al. used classical affinity purification approaches to isolate the σ_2_R binding site and characterized it as the (ER)-resident membrane protein, transmembrane protein 97 (TMEM97), with a molecular weight of about 18–21.5 kDa (frequently referred to as σ_2_R/TMEM97).^26^ In 2011, Xu and co-workers reported that progesterone membrane binding component-1 (PGRMC1) could bind to σ_2_R, altering the pharmacological properties of its ligands.^27^ More recently Mach and collaborators from the University of Pennsylvania, using a gene-editing approach, demonstrated that TMEM97 and PGRMC1 could form a ternary complex with the low density lipoprotein (LDL) receptor leading to a much-increased LDL internalization.^28^ This observation, confirmed by confocal microscopy and radioligand binding studies, indicated the involvement of σ_2_R/TMEM97 in lipoprotein trafficking and could rationalize the upregulation of σ_2_Rs in certain types of cancer cells.^45^

The recent discovery of the identity of the σ_2_R rationalizes the search for small molecules with potential neuroprotective,^29^ antinociceptive,^30^ and antiproliferative effects.^31^ Concerning the role of σ_2_Rs in the context of cancer, different pharmacological studies have proved that the σ_2_R is overexpressed in cancer cells, and its abundance is correlated with the proliferative status of certain tumors.^32^ Furthermore, in addition to the diagnostic imaging application, σ_2_R ligands have shown cytotoxic effects in tumor cells *in vitro* and *in vivo*.^33−36^ A better elucidation of the implication of σ_2_Rs in tumor cell death was reported in 2019 by Zeng and co-workers, which conducted CRISPR/Cas9 studies to assess the cytotoxic properties of σ_2_R ligands in TMEM97 knockout (KO), PGRMC1 KO, or TMEM97/PGRMC1 double KO cell lines.^37^ Results showed that induction of cell death by σ_2_R ligands was not hampered, suggesting that the cytotoxic effects are not directly mediated by TMEM97 or PGRMC1, thus questioning the exact cytotoxic mechanism exerted by σ_2_R ligands.

**Figure 2:**
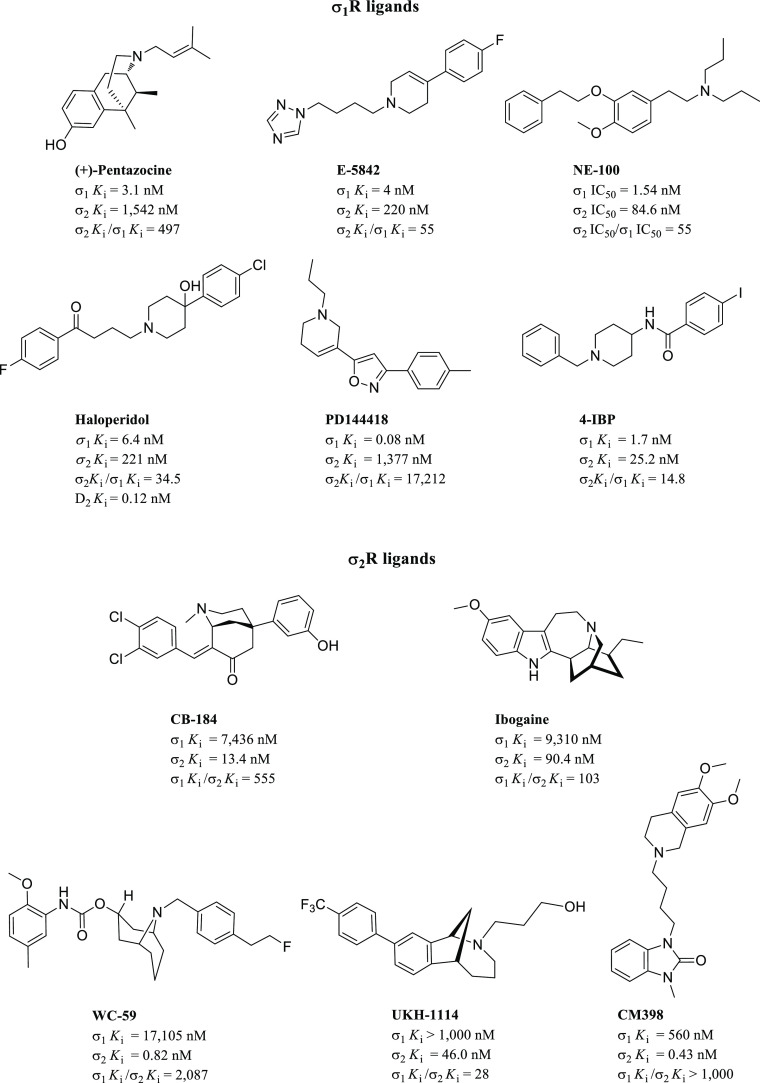
Selected historic and representative σR ligands.

Following the differentiation of the two σR subtypes, tremendous efforts were directed toward developing selective ligands for each subtype. (+)-Pentazocine (Figure 2), the first σ_1_R selective ligand, exhibited 500-fold selectivity (σ_2_*K*_i_/σ_1_*K*_i_) over the σ_2_R receptor.^38^ Also, the 1,2,4-triazole derivative E-5842 (Figure 2) showed a *K*_i_ value of 4 nM for the σ_1_R and 55-fold selectivity.^39^ Later, a dipropylamine derivative named NE-100 (Figure 2) was reported to have a high affinity for the σ_1_R and moderate selectivity over the σ_2_R.^40^ Haloperidol (Figure 2) is a butyrophenone derivative belonging to the drug class of neuroleptics, mainly acting as a D_2_ antagonist. For many years, haloperidol has been used as a reference σ_1_R antagonist, and it represents a classic σR ligand prototype. Its antagonist profile toward the σ_1_R was discovered more than 20 years ago, along with its *in vitro* and *in vivo* anticancer properties toward several cancer types.^41−47^ In 2011, Schläger et al. reported a series of spirocyclic pyranopyrazoles with high σ_1_R affinity and selectivity toward the σ_2_R, α_1_, α_2_, 5-HT_1A_R, and the 5-HT-transporter.^48^ Two chemically and pharmacologically distinct high-affinity σ_1_R ligands named 4-IBP (agonist or inverse agonist) and PD144418 (antagonist) were used to obtain the above-mentioned first crystal structures of the human σ_1_R (Figure 2).^49,50^

Compound CB-184 (Figure 2) was the first reported highly selective σ_2_R ligand back in 1995 by Bowen and co-workers.^51^ This compound was followed by several other σ_2_R ligands belonging to diverse chemical classes (discussed in more detail in Section 3.2), including the indole alkaloid ibogaine^52,53^ and the granatane derivative WC-59.^54^ Most of the reported σ_2_R ligands discovered to date were developed for their cytotoxicity properties toward several cancer cell lines;^43^ however, the most recently reported methanobenzazocine derivative UKH-1114^55^ and the 6,7-dimethoxy-1,2,3,4-tetrahydroisoquinoline derivative CM398^56^ (Figure 2) showed an exquisite σ_2_R selectivity and demonstrated to produce antinociceptive effects *in vivo*.

**Figure 3:**
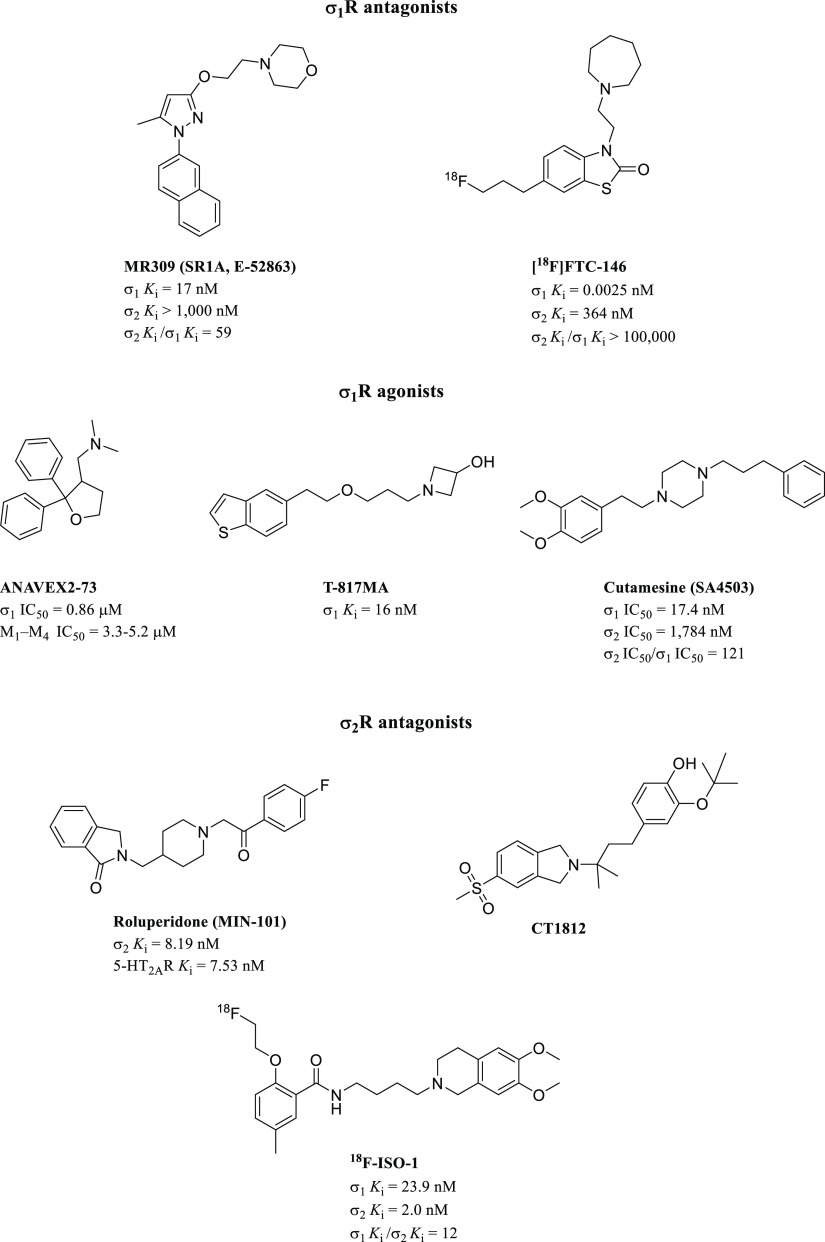
σRs ligands in clinical trials.

To date, a few σR ligands have entered clinical trials to treat different diseases, including neurodegenerative diseases, mental disorders, and pain management. Among them, MR309 (Figure 3) has been the first selective σ_1_R antagonist to reach phase II clinical trials for the treatment of oxaliplatin-induced neuropathic pain, and it represents a potential first-in-class analgesic. The randomized, double-blind, placebo-controlled study started in patients with colorectal cancer receiving FOLFOX, aimed to assess the efficacy of MR309 in ameliorating oxaliplatin-induced peripheral neuropathy (OXAIPN). Interestingly, discontinuous MR309 administration resulted in a potential neuroprotective role for chronic cumulative OXAIPN, with a reasonable safety profile.^57^

The radio-tracer [^18^F]FTC-146 (Figure 3), which is the most selective σ_1_R ligand known to date (>146,000-fold selectivity over the σ_2_R and >10,000-fold selectivity over 59 different targets), is currently in phase I clinical trials as a first-in-class diagnostic agent for positron emission tomography–magnetic resonance imaging (PET–MRI) to detect sites of nerve damage in patients with neuropathic pain.^58^ The nonradiolabeled analog, named CM304 and acting as σ_1_R antagonist, showed a low pharmacokinetic profile with a short *in vivo* half-life (115 min) and undesirable clearance (Cl = 33 mL/min/kg)^59,60^ which did not allow the compound to move into clinical research, even though it showed efficacy in multiple preclinical mice models of pain.^61,62^

The tetrahydrofuran derivative ANAVEX2-73 (Figure 3), acting as a mixed muscarinic receptor/σ_1_R ligand, is currently in phase II clinical evaluation to treat patients with mild to moderate Alzheimer’s disease.^63^ Similarly, T-817MA, a high-affinity σ_1_R agonist with neuroprotective properties in rats,^64^ reached phase II clinic studies for the same medical condition, while cutamesine (Figure 3), another selective σ_1_R agonist,^65^ has been evaluated in phase II studies in patients for recovery enhancement after acute ischemic stroke.^66^

Concerning clinical candidates targeting the σ_2_R subtype, two different antagonists, roluperidone and CT1812 (Figure 3), entered phase II and phase I clinical trials to establish their efficacy and safety in the treatment of schizophrenia and Alzheimer’s disease, respectively.^67,68^ Interestingly, none of the σR ligands with intrinsic cytotoxicity properties discovered so far are in clinical trials to treat cancer, likely due to the inconsistent data concerning the efficacy of σRs ligands on preclinical *in vivo* models. This situation was aggravated by the unavailability of the genetic data of the σ_2_R subtype until its cloning in 2017. Usually, full characterization of the molecular target is required to link the chemical probe-target engagement to the functional pharmacology before launching a full drug discovery program. Indeed, the precise role of σRs in cancer biology has not yet been entirely clarified. However, the involvement of σRs in the induction or inhibition of apoptosis, cell growth, proliferation, and tumor progression paved the way for developing small molecules that could be exploited in novel anticancer therapies. The cytotoxic or antiproliferative properties of σR modulators are exerted by interfering with both σ_1_ and σ_2_ receptors. In particular, inhibition of the σ_1_R or induction of the σ_2_R activities seems to lead to tumor growth inhibition.^69^ Specifically, it has been observed that σ_1_R negative modulators cause a caspase-dependent induction of apoptosis, whereas σ_2_R positive modulators mediate a caspase-independent induction of programmed cell death,^70−72^ even though this aspect could not be considered as a rule of thumb because of some exceptions. Generally, a reliable *in vitro* protocol useful to distinguish between the agonist or antagonist properties of σ_1_R and σ_2_R ligands has not been established yet, mostly due to a lack of known endogenous ligands which does not allow to compare the molecular effect of a tested compound at the level of the receptor. Despite this fact, the apoptotic mechanism of induction employed by σR modulators has been previously used as a judgment parameter to establish the functional activity of σ_2_R ligands, as described by Zeng et al. in 2014.^73^ However, based on the recent finding reported from the same research group, nowadays it is known that this approach of σR ligands characterization is not suitable.^37^

Nevertheless, the selective overexpression of σR in cancer cells makes it an attractive target for developing useful diagnostic agents, such as the σ_2_R molecular probe named ^18^F-ISO-1 (Figure 3), that has been assessed in clinical trials to evaluate the safety and feasibility of imaging tumor proliferation by PET in patients with diagnosed malignant tumors.^74,75^ Regarding this aspect, several comprehensive review articles dealing with the development of σR radiotracers to diagnose cancer have been recently published;^76−78^ thus, we will not discuss this further. Alternatively, discovering novel anticancer agents that can potentially treat certain tumors with a more selective cytotoxic profile would significantly advance global health.

Based on these premises, this perspective highlights state of the art development of σR ligands with potential anticancer activity, mainly covering articles published between 2010 and 2020. In particular, in this work, the literature search has been conducted using SciFinder and PubMed online databases and choosing “sigma receptors, sigma-1 ligands, sigma-2 ligands, cancer, cytotoxicity, anticancer agents” as keywords. After a cross-match searching process, only significant research articles strictly related to this perspective’s topics have been selected. First, we will briefly overview the most significant σR pharmacophore models reported to date to provide detailed information about essential chemical features required for ligands binding at σRs. Second, the most recently reported σR ligands with antiproliferative and cytotoxic activities will be covered, particularly selective σ_1_R and σ_2_R ligands. Extensive structure–affinity relationships (SAfiRs) and structure–activity relationships (SARs) regarding the main classes of σR ligands will be discussed and summarized in each section. Finally, a comprehensive medicinal chemistry perspective on the past, present, and future of σR ligands as a new potential generation of cytotoxic agents will be provided.

## 2 Pharmacophore Models for σR Ligands

**Figure 4:**
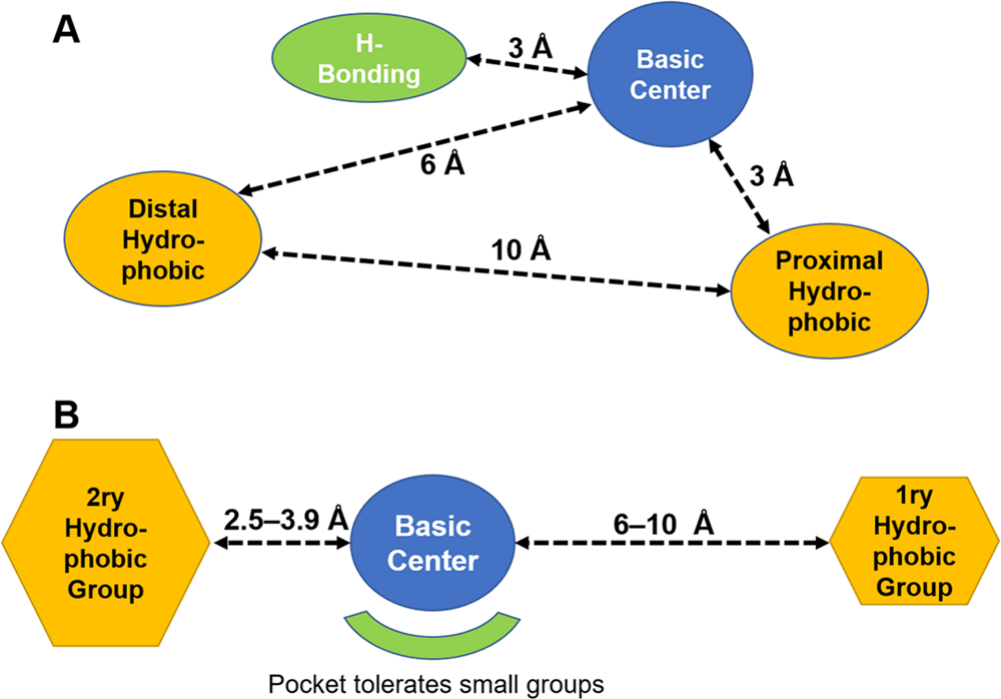
(A) The proposed pharmacophore model by Gilligan et al. (B) Glennon’s pharmacophore model.

There is a large number of reported σR ligands in literature with no clear SAfiRs or SARs. As mentioned earlier, the crystal structure of σ_1_R was released in 2016. Also, the 3D structure of σ_2_R is not yet available for structure-based design. Hence, most of the rational^57,79^ design attempts of σR ligands were ligand-based modeling. The main issue that hindered the development of pharmacophore models for σRs is the structural diversity of the reported ligands. The first model was reported by Gilligan et al. in 1992 with four pharmacophore elements for σ_1_R binding, namely, a basic nitrogen atom, two hydrophobic groups, and an H-bonding center midway between the basic N and the distal hydrophobic site (Figure 4A).^80^ It is worth noting that this first model was not ideal for database mining since it only explained the binding characteristic of one class of compounds.

Following that early attempt by Gilligan, several ligand-based models for σ_1_R or σ_2_R ligands have been reported. These models and their applications will be discussed in the following section. The second prominent attempt was reported by Glennon and co-workers in 1998. They developed a comparative molecular field analysis (CoMFA) model based on the binding data of 64 benzonorbornane derivatives to σ_1_R. The model showed a good correlation coefficient (*R*^2^ = 0.989) and predictive ability (*Q*^2^ = 0.732) and supported the proposal of Gilligan. The Glennon model comprises a central basic nitrogen flanked by two hydrophobic/aromatic moieties, one of them is significantly larger than the other (Figure 4B). Also, the hydrophobic groups are not located at equal distances from the basic core. The smaller is 2.5–3.9 Å, while the larger group is proposed to be 6–10 Å away from the basic nitrogen (Figure 4B). One of the early trials to address the diversity of σ_1_R ligands in the developed pharmacophore was reported by Jung and co-workers in 2004. This model includes two aromatic rings, a carbon centroid, the basic nitrogen, and a hydrogen bond close to the basic nitrogen (not shown). In 2009, Glennon reported a CoMFA model for σ_2_R based on a series of cyclohexylpiperazines. This model showed a similar arrangement to previously reported σ_1_R models with a correlation coefficient of 0.95 and a cross-validated one of 0.73.^81^

**Figure 5:**
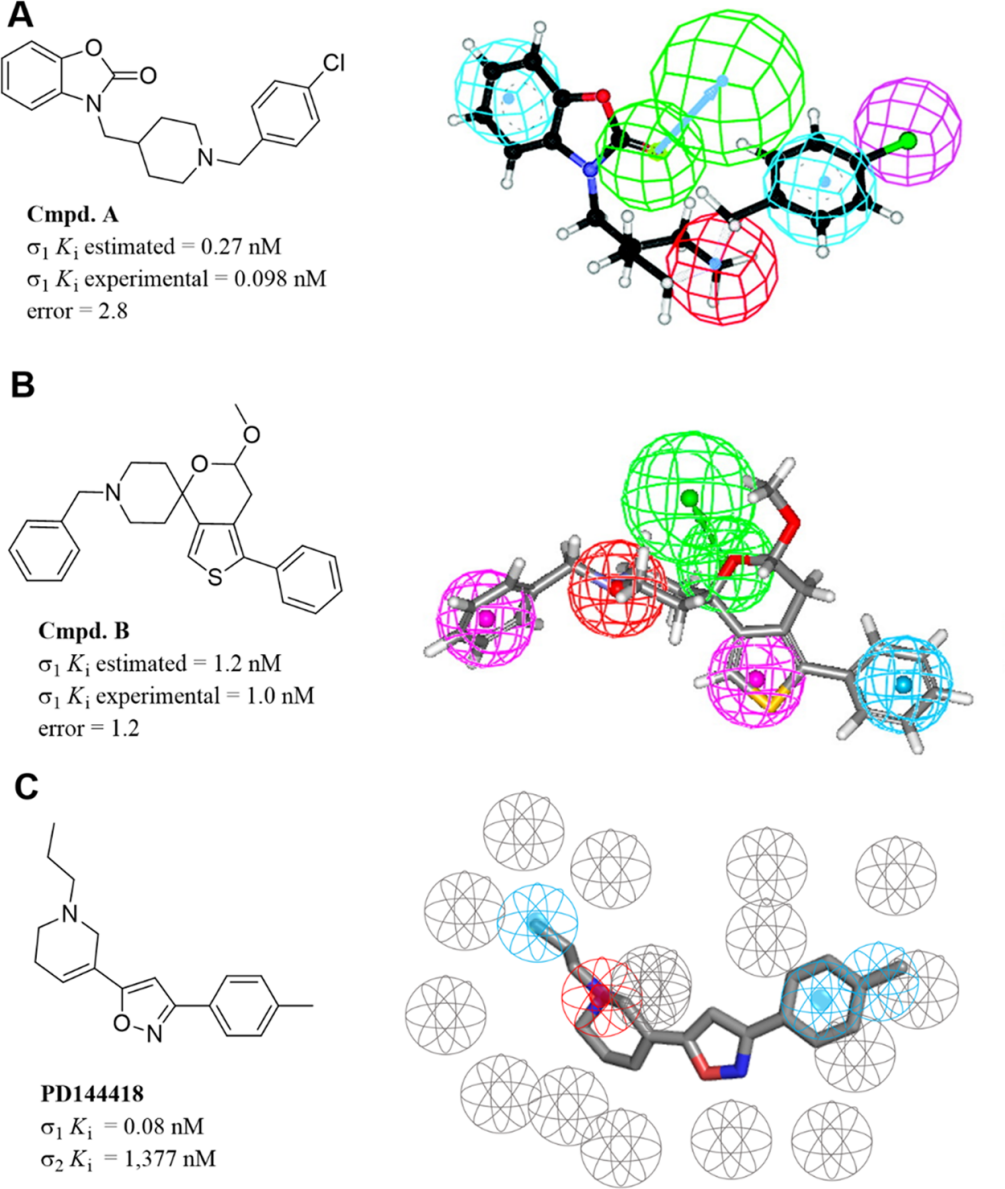
3D pharmacophore models for σ_1_R: (A) Pharmacophore mapping of compound **A** in 3D models derived by Vio et al. (B) Pharmacophore mapping of compound **B** in 3D models derived by Meyer et al. (C) Pharmacophore mapping of PD144418 in 3D models derived by ESTEVE. Color coded as follows: PI (red), HYAr or HYD (light blue), HY (pink), HBA (light green), excluded volumes (gray). Adapted with permission from refs (82 and 83). Copyright 2009 and 2012 American Chemical Society.

Vio et al. used Catalyst software and the HypoGen algorithm to prepare a common-feature pharmacophore for a series of benzo[*d*]oxazolone with high affinity to the σ_1_R.^82^ Although the model was based on different compound series, it was in perfect agreement with Glennon’s previous model with very similar distances and features, including two aromatic rings (HYAr), one hydrophobic (HY), one hydrogen-bond-acceptor group (HBA), and one positive ionizable (PI) feature (Figure 5A).

**Figure 6:**
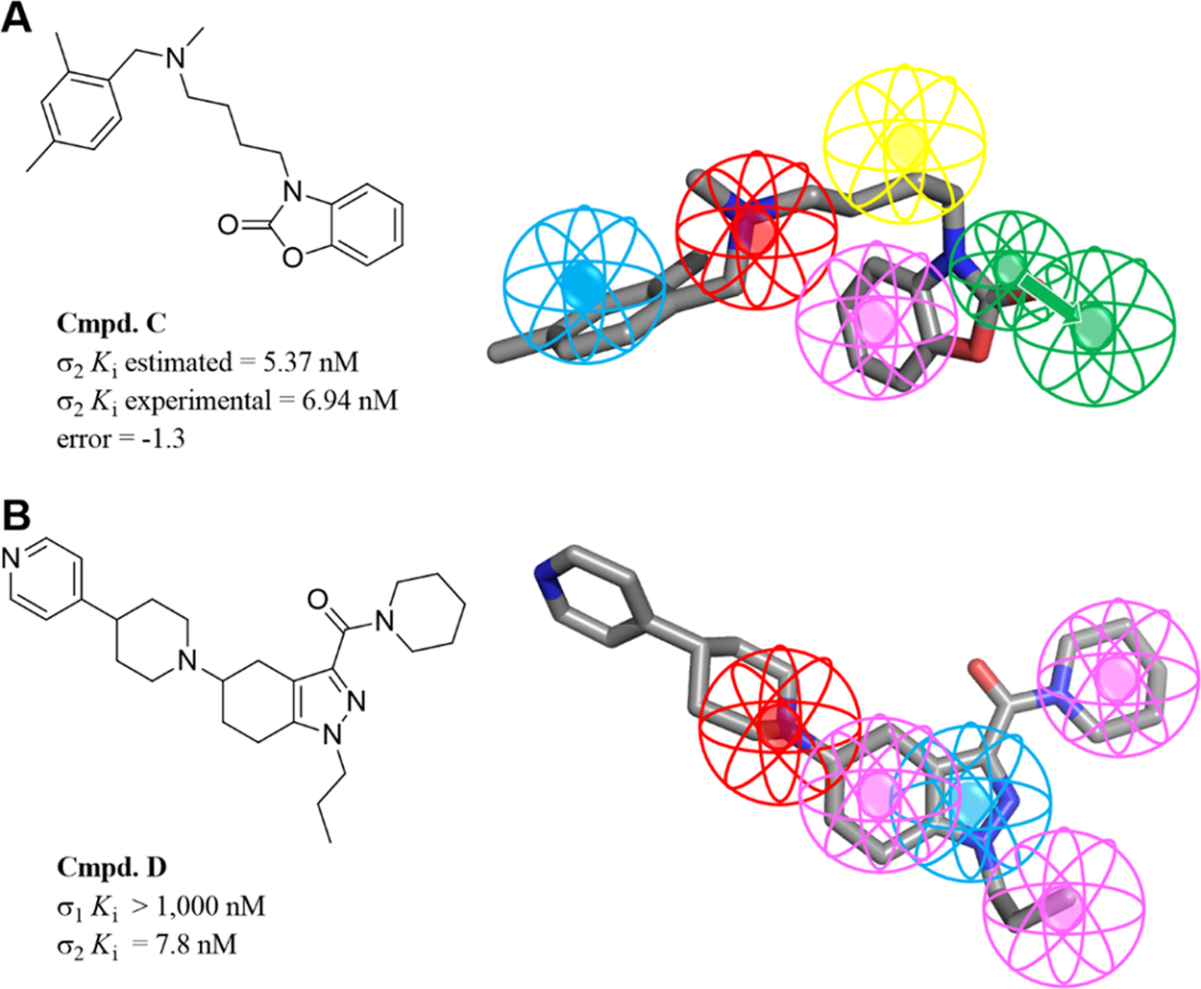
3D pharmacophore models for σ_2_R: (A) Pharmacophore mapping of compound **C** in 3D models derived by Vio et al. (B) Pharmacophore mapping of compound **D** in 3D models derived by Iyamu et al. Color coded as follows: PI (red), HYAr or HYD (light blue), HY (pink), HBA (light green), excluded volumes (gray).

The same research group has also reported another pharmacophore model for σ_2_R ligands based on benzo[*d*]oxazolone derivatives (Figure 6A).^84^ The latter exhibited a very similar arrangement to that of the σ_1_R previously developed by the same group and the one developed by Glennon. Nevertheless, compared to Glennon’s pharmacophore, the distance between the primary hydrophobic region and the basic nitrogen is significantly shorter (4.96 Å).^84^

In 2012, Meyer et al. reported a σ_1_R ligands pharmacophore based on a novel series of spirocyclic thiophenes also using the Catalyst software (Figure 5B).^83^ This model is comprised of the same five features described by Vio et al. with the positive ionizable feature located 4.36 and 9.77 Å away from the two hydrophobic features. Recently, in 2019, scientists from the ESTEVE pharmaceutical company developed a σ_1_R pharmacophore based on the published crystal structure (PDB ID: 5HK1) of the receptor (Figure 5C).^85^ This model was compared to the previously mentioned pharmacophores and has been validated using a large database of 25,000 compounds with known σ_1_R affinity. Researchers have used the receptor–ligand pharmacophore generator job implemented in the Discovery Studio software which identifies all the critical ligand–protein interactions and then places exclusion volumes to account for any steric considerations. This model identified Glu172 as the positive ionizable group site while placing the two hydrophobic features within the space defined by residues Tyr103, Leu105, Leu95, Tyr206, Leu182, and Ala185 and confined by helices α4 and α5. This ESTEVE pharmacophore outperformed previously published models and, according to the authors, showed better results than molecular docking. On the other hand, the most recent σ_2_R ligands pharmacophore was published in 2019 by scientists from Northwestern University.^86^ They developed a series of tetrahydroindazoles and used the obtained SAR data to build the model using the PHASE module as implemented in the Schrödinger software suite. This pharmacophore model consists of one PI group, one HYAr ring, and three HY moieties (Figure 6B). Although the distances were not reported, the arrangement showed an equal disposition between the basic nitrogen and two hydrophobic groups.

**Figure 7:**
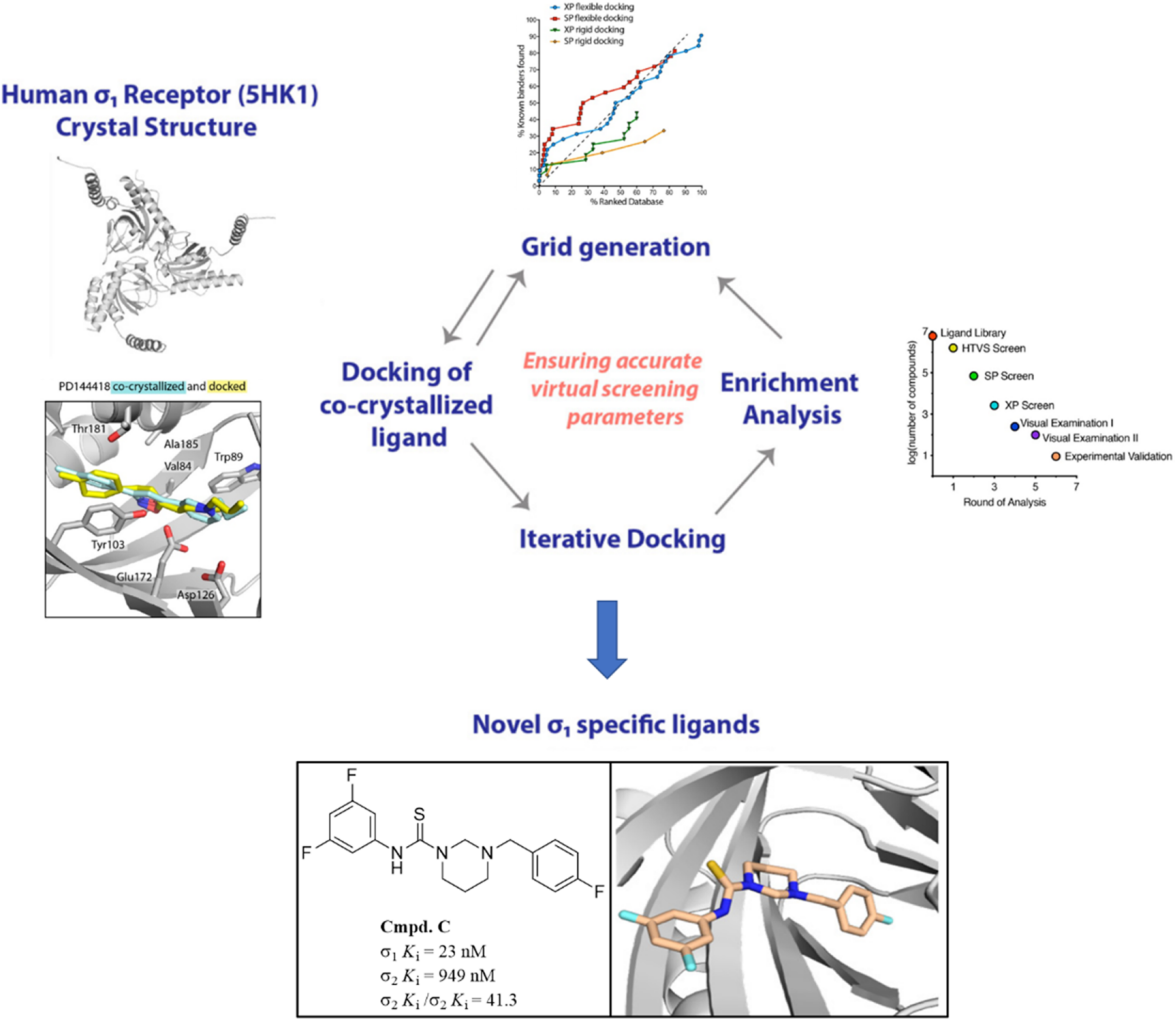
Schematized representation of the high-throughput structure-based computational docking approach for the discovery of new σ_1_R ligands proposed by Greenfield et al. Adapted with permission from ref (90). Copyright 2020 American Chemical Society.

Generally speaking, the development of σ_1_R ligands depended mainly on ligand-based design, especially on the general pharmacophoric features suggested by Glennon and co-workers.^87,88^ Despite the availability of the σ_1_R crystal structure since 2016, several studies still depend on the general sigma pharmacophore for the design of novel ligands. This approach has been successful and led to the discovery of several high affinity ligands.^82,85,89^ Moreover, some of the reported pharmacophore models addressed the σR subtype selectivity and highlighted the feature required for binding at each subtype. However, to date, we believe that selectivity against other CNS receptors has not been adequately addressed using ligand-based design methods. Noteworthy, Greenfield et al. recently provided a good example of a high-throughput structure-based computational docking approach as an effective method for the discovery of new selective σ_1_R ligands (Figure 7).^90^ The platform has been developed performing an iterative process of molecular docking experiments with increased precision levels through screening a library of 6 million compounds.

It should be emphasized that most of the known σR ligands possess heterogeneous structures and can adopt several binding conformations targeting any of the σR subtypes. Developing a 3D QSAR model covering diverse ligands adapting various binding modes is challenging. Also, the binding site of the crystallized σ_1_R is flexible, elongated, and can bind large diverse molecules.^20,21^ Docking into such flexible sites is difficult and could give a high percentage of “false positives”. Therefore, we generally recommend using a combined ligand-based (pharmacophore or QSAR) and structure-based (homology modeling and docking) approach. A combination of drug design methods should better predict the activity and eliminate more of the “false positives”.^91^

## 3 σR Ligands with Cytotoxic Effects

### 3.1 Selective σ_1_R Ligands

**Figure 8:**
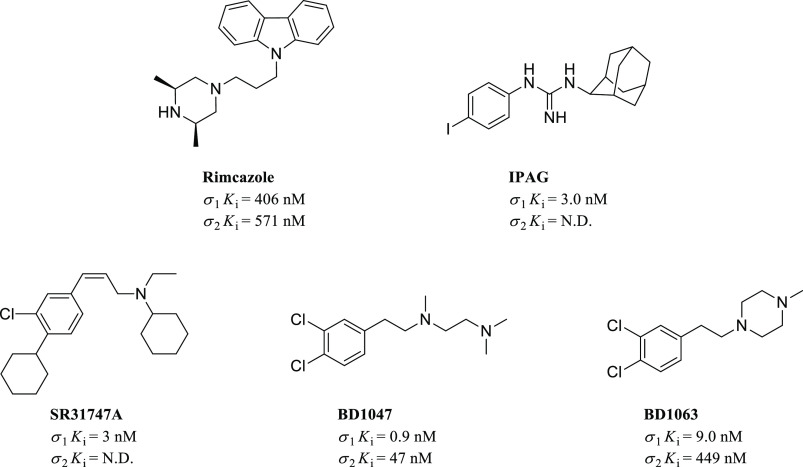
Representative structures of antiproliferative σ_1_R ligands with their σRs binding profile.

Over the years, several selective σ_1_R ligands whose antiproliferative properties have been corroborated by several studies have been reported. Figure 8 shows chemical structures and σRs binding profiles of a few examples of such σ_1_R ligands.

Rimcazole binds to σRs, serotonin transporter, and with higher affinity to the dopamine transporter.^92^ It was initially evaluated for the treatment of schizophrenia and later for its anticancer activity. Specifically, the antiproliferative effects exerted by rimcazole are counterbalanced by the σ_1_R agonist (+)-SKF-10047; thus, rimcazole has been classified as a putative σ_1_R antagonist.^69^ Moreover, rimcazole demonstrated to inhibit cell proliferation on xenografted models of hormone-sensitive and insensitive breast cancer cell lines.^70,93^ Particularly, completion of its anticancer activity seems to require the HIF-1α induction (mediator of apoptosis)^94^ or the presence of the p53 protein.^69^ Despite these preclinical results, rimcazole was never fully considered a potential candidate for anticancer therapy because of its off-target effects related to the interference with dopamine neurotransmission. IPAG, initially synthesized as a possible radiotracer, is a potent tumor suppressor and autophagy inducer.^95^ Also, its ability to induce an unfolded protein response has been reported in several carcinomas, including pancreas and prostate cancers.^95^ SR31747A is a selective σ_1_R antagonist whose antiproliferative activity is associated with immune-modulatory effects in different cancer cell lines with tumor growth inhibition values ranging from 40% to 60% based on the tumor cell lines tested.^96,97^ Finally, the arylalkylethylenediamines BD1047 and BD1063 represent two putative σ_1_R antagonists devoid of cytotoxic properties, although they can induce an altered cell morphology. In general, BD1047 has shown better antitumor effects when compared to its piperazine derivative BD1063.^70,10^

#### 3.1.1 *N*,*N*-Dialkyl and *N*-Alkyl-*N*-aralkyl Fenpropimorph Derivatives

**Figure 9:**
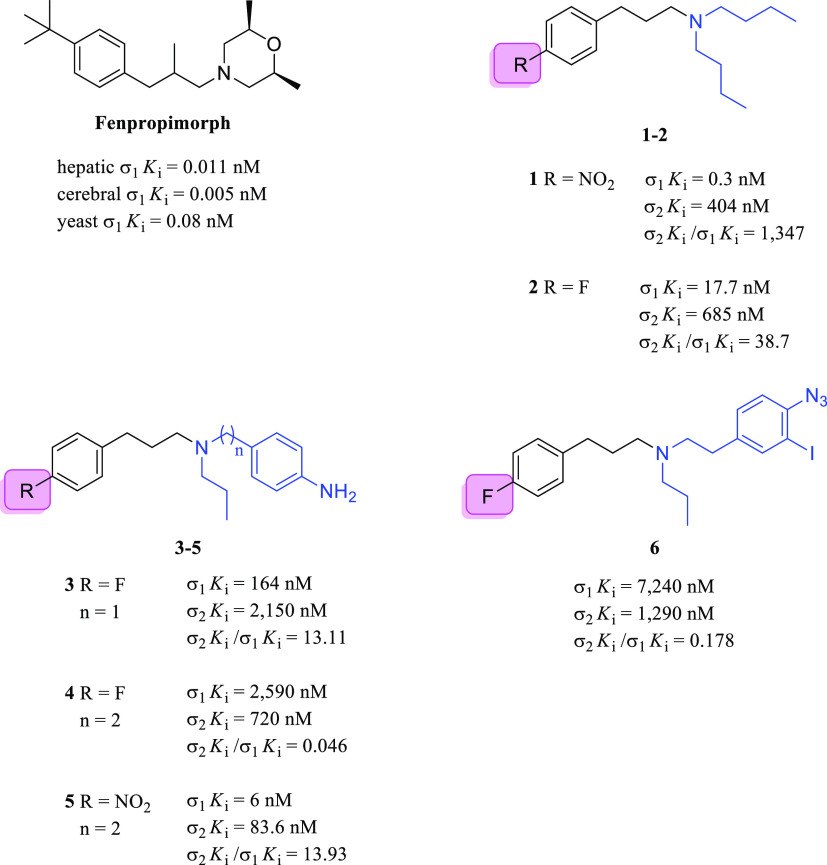
*N*,*N*-Dialkyl and *N*-alkyl-*N*-aralkyl fenpropimorph-derived compounds **1**–**6** and their σRs binding profile.

Despite its amino acid sequence similarity with the yeast C8–C7 isomerase, in 1996, the Glossman research group proved that σ_1_R is devoid of any sterol isomerase activity.^18^ Later, the same team found that fenpropimorph (an agricultural fungicide whose mechanism of action implies disruption of ergosterol biosynthesis pathways) had a high affinity for σ_1_Rs (Figure 9). In 2007, Ramachandran and colleagues purified a recombinant guinea pig σ_1_R and identified two regions, named steroid binding domain-like I and steroid binding domain-like II, that can serve as additional σ_1_R binding sites.^98−100^ Interestingly, these regions share a high similarity with that of the sterol binding domains of the yeast sterol C8–C7 isomerase. The fenpropimorph’s chemical structure consists of an aryl ring linked through an alkyl spacer to a nitrogen atom incorporated in a morpholine ring. Considering that the pharmacophore of σ_1_R ligands and the chemical scaffold of fenpropimorph are superimposable, in 2010, Hajipour and co-workers reported the synthesis of *N*,*N*-dialkyl and *N*-alkyl-*N*-aralkyl fenpropimorph-derived compounds as σR ligands with cytotoxic properties.^101^ Among all the tested compounds, **1**–**3** and **5** exhibited high affinity for the σ_1_R subtype with *K*_i_ values in the nanomolar range. On the contrary, compounds **4** and **6** displayed a slight preference for the σ_2_R subtype (Figure 9), even with σ_2_*K*_i_ values in the high nanomolar or micromolar range.

SARs were defined for this series of compounds. In general, compounds with a nitro substituent on the phenyl ring were more potent than the corresponding fluorinated derivatives. Compound **1** was about 59-fold more potent than compound **2**, whereas compound **5** was 400-fold more potent than its fluorinated analog **4**. The authors suggested that the electron-withdrawing properties of the nitro group presumably enhanced the binding with the σ_1_R. Interestingly, when a fluorine atom is present (e.g., compound **4**), the binding properties changed in favor of the σ_2_R subtype. The authors also reported the importance of the free lone pair of the nitrogen atom on the alkyl chain, which was necessary for the binding with the σ_1_R, according to Glennon et al.^102^ Indeed, the amide derivative of compounds **2** (not shown) did not have any affinity for the σ_1_R. Compounds **1**–**6** were tested for their cytotoxic properties against a broad panel of tumor cell lines (Table 1).

**Table 1:** IC_50_ Values of Compounds **1**–**6** on a Selected Panel of Cancer Cell Lines

	IC_50_ (μM)^a^									
Compd.	NCI-H460	H1299	SKOV-3	DU145	MCF7	MCF10A	MB-MDA-231	SF268	HT-29	HCT-15
**1**	40.52	>100	27.85	32.67	22.36	>100	57.12	>100	>100	>100
**2**	>100	>100	56.18	>100	>100	>100	>100	>100	>100	>100
**3**	>100	>100	>100	>100	>100	>100	>100	>100	>100	>100
**4**	44.77	>100	20.15	>100	41.34	>100	68.12	>100	>100	>100
**5**	>100	>100	>100	>100	88.1	>100	>100	>100	>100	>100
**6**	40.32	90.81	>100	13.06	16.75	88.63	21.60	38.8	36.42	54.12

a Data from ref (101).

Compound **1** showed activity against NCI-H460, SKOV-3, DU145, MCF7, and MB-MDA-231 cancer cell lines. On the other hand, compound **2**, which differs from **1** only for the fluorine atom, was active only against SKOV-3 with an IC_50_ value of 56.18 μM. Compound **5** demonstrated moderate activity against the MCF7 cancer cell line with an IC_50_ value of 88.1 μM. Interestingly, an opposite trend was observed for compound **4**, the fluorine derivative of **5**. In fact, the former was found to be active on NCI-H460, SKOV-3, MCF7, and MB-MDA-231 tumor cell lines. The better affinity of compound **4** for the σ_2_R subtype with respect to the σ_1_R seems to explain this behavior. Indeed, the σ_2_R subtype is overexpressed in different cancer cell lines, and the authors, at the time of the publication, were not able to explain if the cytotoxic activity was due only to the involvement of the σ_2_R or both receptors. Compound **3** showed to be devoid of any cytotoxic activity in all the tested cell lines. Finally, compound **6** showed no specific cytotoxicity in all the selected cancer cell lines, except for SKOV-3.

#### 3.1.2 Spirocyclic Piperidine Derivatives

##### 3.1.2.1 Spipethiane Derivatives

**Figure 10:**
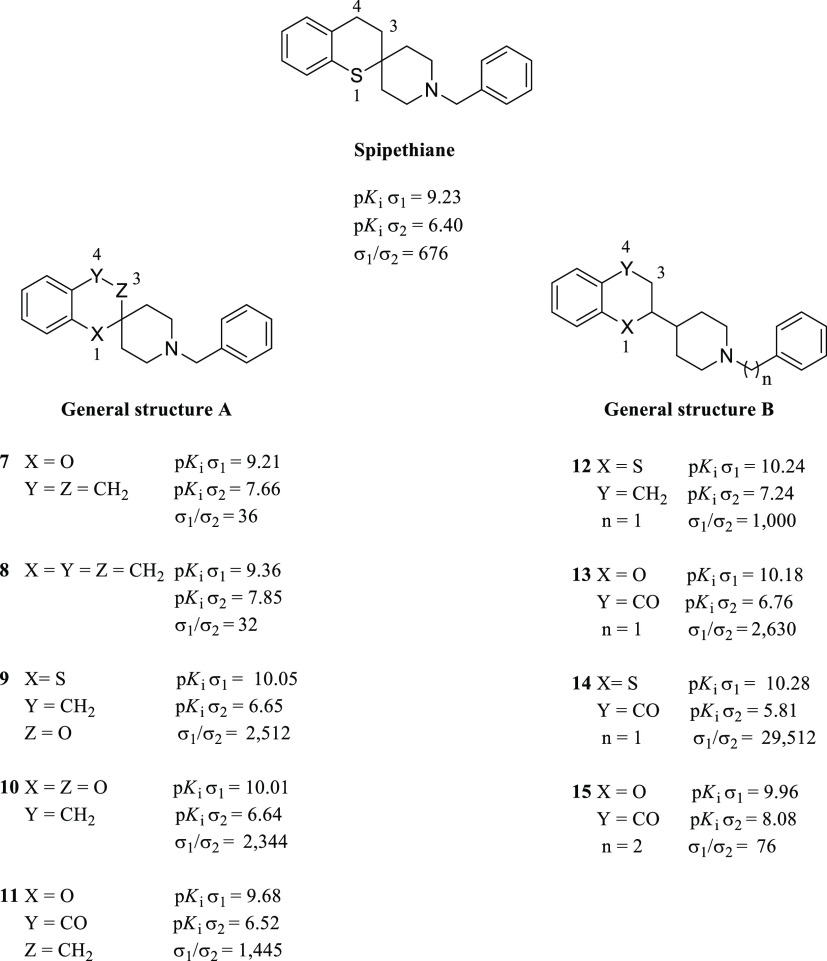
Spipethiane and general structure of spipethiane derivatives **7**–**11** and **12**–**15** with their σRs binding profile.

In 2010, Piergentili et al. discovered novel highly potent and cytotoxic σ_1_R ligands with a putative antagonistic profile whose chemical structure was based on the spipethiane scaffold (σ_1_ p*K*_i_ = 9.23, σ_2_ p*K*_i_ = 6.40, Figure 8),^103^ a spiro compound identified as a σR ligand by the same research group in 1998. Bioisosteric substitutions of the sulfur in position 1 and the methylene group in position 3 of spipethiane were performed to expand the SARs of this class of compounds (general structure A, compounds **7**–**11**, Figure 10). In addition, a smaller second set of compounds was obtained by deleting the spiro carbon and separating the two hydrophobic portions of the molecule through a carbon–carbon single bond (general structure B, compounds **12**–**15**, Figure 10). Moreover, insertion of a carbonyl function in position 4 and homologation at the nitrogen atom of the piperidine ring was also considered. Based on σ_1_ and σ_2_ radioligand binding assays performed, respectively, on Jurkat cells and rat cerebral cortex membranes using [^3^H]-pentazocine and [^3^H]1,3-di(2-tolyl)guanidine ([^3^H]DTG) as labels, SARs were built up. Almost all the novel compounds of the two series had higher σ_1_R affinity values than the lead spipethiane. Bioisosteric substitution of the sulfur atom with oxygen or with a methylene group did not alter the σ_1_R affinity even if a slight increase of affinity was considerable for the σ_2_R (compounds **7**–**8**) with a consequent decrease of σ_1_*K*_i_/σ_2_*K*_i_ selectivity ratio (calculated as the antilogarithm of the difference between p*K*_i_ at σ_1_ and σ_2_ receptors). Better results were obtained with compounds **9** and **10**, where the methylene group of spipethiane in position 3 was substituted with an oxygen atom. Interestingly, replacement of the methylene group in position 4 with a ketone functional group afforded the best compounds in terms of σ_1_R affinity. The best result was obtained with compound **14**, also characterized by the absence of the spiro carbon that links the thiochromane ring to the benzylpiperidine moiety. This compound possessed a σ_1_ p*K*_i_ of 10.28 (σ_1_*K*_i_/σ_2_*K*_i_ = 29,512), and it represented the best σ_1_R ligand reported in the literature at the time of the publication. The high affinity of **14** was explained by hypothesizing that its more flexible structure allowed better interactions with the σ_1_R binding site. However, an increase in flexibility is not always a rule of thumb to be applied to develop selective σ_1_R antagonists. Indeed, compound **15**, which is the compound **13** homologous at the nitrogen atom of the piperazine ring, had a lower σ_1_ p*K*_i_ = 9.96 and a σ_2_ p*K*_i_ = 8.08. Therefore, it seemed that the elongation of the alkyl chain in this class of compounds ameliorated the σ_2_R affinity and caused a drastic loss of σ_1_*K*_i_/σ_2_*K*_i_ selectivity ratio.

The spipethiane derivatives **7**–**15** were tested on MCF-7 and MCF-7/ADR cancer cell lines to evaluate their antitumor properties. The two cancer cell lines were chosen on the basis of their differential expression of σRs; in particular, the high overexpression of σ_1_R subtype characterizes the latter.^104^ The authors proved that all the novel compounds possessed cytostatic properties in the MCF-7/ADR cancer cell line with the best GI_50_ values obtained with compounds **11** and **13** (10.0 μM and 7.7 μM, respectively), whereas no growth inhibition was observed in the MCF-7 cancer cell line. Also, an analysis of the ability to interfere with the cell cycle by comparing the spipethiane and compounds **13** and **14**, which possessed the best σ_1_*K*_i_/σ_2_*K*_i_ selectivity ratio, was made. Specifically, compounds **13** and **14** increased the number of cells in the G_0_/G_1_ phase and decreased the number of cells in the S phase in the MCF-7/ADR cell line; the same trend was not observed in MCF-7 breast cancer cells. Contrariwise, spipethiane was not able to affect the cell cycle. The capability of compounds **13** and **14** to induce apoptosis was also described. Indeed, MCF-7 and MCF-7/ADR cancer cell lines were stained with annexin V-FITC to evaluate the expression of phosphatidylserine on the outer layer of the cell membrane, which represents a typical feature of cells in apoptosis. Flow cytometry analysis highlighted phosphatidylserine expression only in MCF-7/ADR cells treated with compounds **13** and **14**. Finally, the functional activity of compound **14** was validated using the tail-flick assay. σ_1_Rs are highly expressed in the dorsal horn of the spinal cord,^105^ and it has been demonstrated that they can modulate opioid analgesia.^106^ In addition, KO of the σ_1_R gene (*SIGMAR1*) determines pain-attenuated phenotype in mice, supporting the modulatory role of σ_1_Rs in different types of pain (e.g., neuropathic, inflammatory, visceral).^107^ Therefore, σ_1_R ligands mimicking this condition are considered putative σ_1_R antagonists or negative modulators. Treatment of CD-1 mice only with **14** did not induce any analgesic effect, whereas pretreatment with morphine and subsequent administration of **14** enhanced the analgesic effect of morphine itself. Altogether, these results were consistent with previously reported findings on σ_1_R antagonist (i.e., BD1047)^106^ and proved the putative σ_1_R antagonist profile of compound **14**. In our opinion, the combination of structural elements, σRs binding profile, and intrinsic activity makes spipethiane derivatives an exciting class of compounds that might be further developed as cytotoxic agents helpful in those cancer cell lines whose aggressiveness is related to the overexpression of σ_1_Rs.

##### 3.1.2.2 Spirocyclic Thienopyran and Thienofuran Derivatives

**Figure 11:**
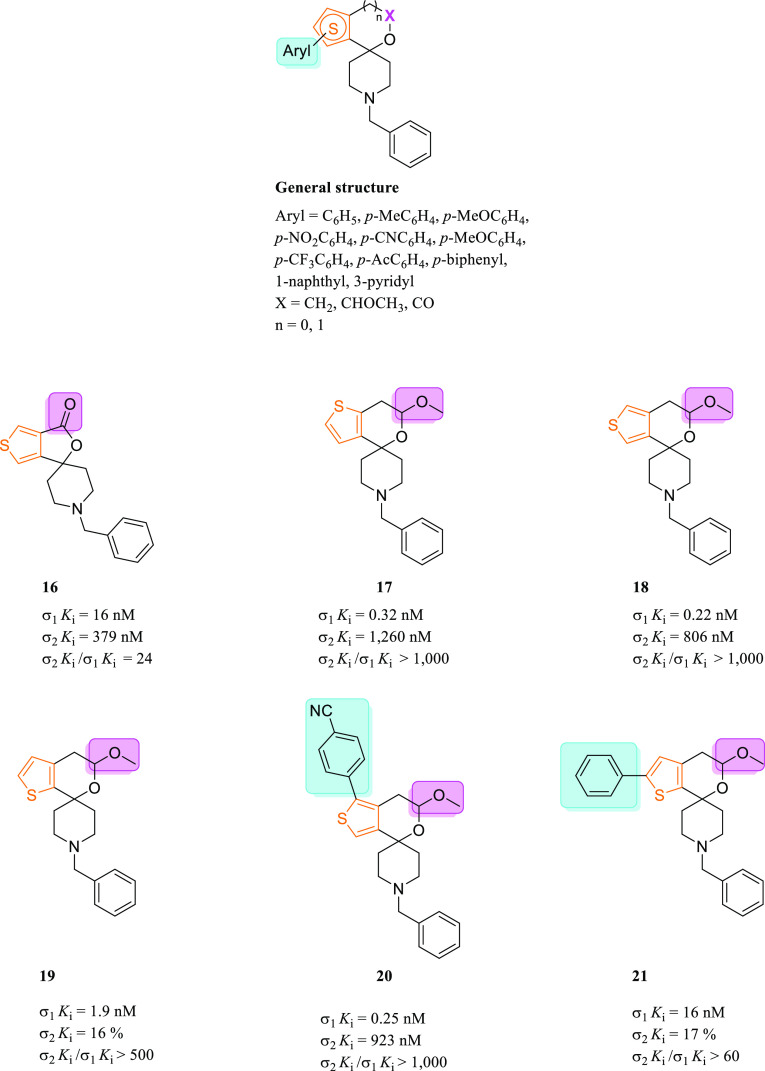
Structures of selected spiropiperidines with a thienofuran and thienopyran scaffold (**16**–**21**) and their σRs binding profile.

In the search for selective σ_1_R ligands, molecules with a spirocyclic piperidine scaffold gained much attention over the last decades. With this in mind, the synthesis of spirotetralins, spiro-joined benzofuran, isobenzofuran, and benzopyran piperidine derivatives were described, along with their affinities toward the two σR subtypes.^108−110^ Bioisosteric substitution of the benzene ring of spiro-joined benzofuran and benzopyran piperidine derivatives with a thiophene ring gave highly potent and selective σ_1_R ligands. Compounds **16**–**21** were identified as the most interesting compounds belonging to this series (Figure 11). The main differences between these spiro-piperidines are (i) the presence of an aryl moiety linked to the α-position of the thiophene ring; (ii) the size of the oxygenated ring; and (iii) the nature of the substituent linked to such ring. Among the non-arylated lactones, a thieno[3,4-*c*]furan-3-one scaffold (**16**) showed the best results in terms of σ_1_R affinity when compared to the pyranone ring (not shown). Arylation of **16** in the 4′-position of the thiophene ring (on the same side of the lactone functional group) with rings possessing electron-withdrawing or electron-donating groups was tolerated. Despite a slight increase of the σ_1_R affinity of such compounds, the non-arylated compound **16** still possessed a better σ_2_*K*_i_/σ_1_*K*_i_ selectivity ratio, so that additional derivatives of **16** substituted in the 4′-position have not been investigated. On the contrary, arylation at the 6′-position of the thiophene ring of **16**, or at both 4′- and 6′-positions, caused loss of affinity for the σ_1_R. Regarding the acetalic spiropiperidines with a thienopyran moiety (**17**–**19**), the sulfur position influenced the σ_1_R affinity. Indeed, the regioisomer **19** with a thieno[2,3-*c*]pyran moiety was about 6-fold and 8.5-fold less potent than regioisomers **17,18** (σ_1_*K*_i_ = 1.9 nM for **19** vs σ_1_*K*_i_ = 0.32 nM and 0.22 nM for **17** and **18**, respectively). α-Arylation of compound **17** with a thieno[3,2-*c*]pyran moiety led to compounds whose σ_1_R affinity is from 17- to 500-fold higher than those of the parent compound **17**.^83^ On the contrary, α-arylation of compound **18** (on the same side of the acetalic function) and α-arylation of compound **19** were tolerated. For derivatives of compound **18**, both electron-rich and electron-poor phenyl rings as well as naphthyl rings were tolerated. However, a bulky biphenyl moiety was not tolerated. Interesting results have been obtained by insertion of a *p*-cyanophenyl substituent on the thiophene ring of compound **18**. This derivative (compound **20**) displayed a σ_1_*K*_i_ value of 0.25 nM, which is perfectly comparable with the σ_1_*K*_i_ value obtained for compound **18** (0.22 nM). Meyer et al., who designed and synthesized these molecules, explained this aspect by a possible interaction of the additional aryl moiety with a hydrophobic pocket within the σ_1_R protein that is not accessible for the non-arylated parent compounds. In light of the σ_1_R values, it is clear that the spirocyclic scaffold was responsible for the high affinity for the σ_1_R. In contrast, the aryl moiety allows only additional hydrophobic interactions useful for better binding with the receptor.^111^ Aryl derivatives of **19** only tolerated unsubstituted or electron-poor aryl substituents (**21**, σ_1_*K*_i_ = 16 nM).^112^ In order to establish the selectivity over other receptors, the affinities of compounds **16**–**21** toward the phencyclidine binding site and the ifenprodil binding site of the NMDA receptor (GluN2B) were assessed. Results showed affinity values exceeding 500 nM and selectivity for the σ_1_R, except for compound **20**, which displayed a *K*_i_ = 91 nM for the GluN2B.^113^ Recently, pharmacological characterization of compounds **16**–**21** has been performed.^113^ Non-arylated compounds **16**–**19** did not display affinities for the serotoninergic 5-HT_1A_ receptor, the adrenergic α_1A_ and α_2A_ receptors, and the serotonin transporter (SERT). Compounds **16**, **20**, and **21** displayed a negligible affinity for opioid receptors, whereas compound **18** showed a moderate affinity for κ-opioid receptor (KOR) and δ-opioid receptor (DOR).

The σ_1_R functional activity of spirocyclic piperidines **16**–**21** was investigated by merging the information acquired from these compounds’ effect on the induced Ca^2+^ influx mediated by voltage-gated channels in retinal ganglion cells and the capsaicin assay. In the first test, a non-arylated and an arylated compounds (**18** and **20**) were used. The KCl-induced Ca^2+^ influx through the L-type voltage-regulated Ca^2+^ channel was inhibited in the presence of a σ_1_R agonist. On the contrary, σ_1_R antagonists had the opposite effect. Both compounds did not inhibit the KCl-induced Ca^2+^ influx, whereas they could reverse the inhibition mediated by the σ_1_R agonist opipramol, suggesting a σ_1_R antagonist profile. The putative σ_1_R antagonist properties were further confirmed with an *in vivo* capsaicin assay, in which compounds **18**, **19**, and **21** exhibited antiallodynic effects and a prolonged response latency after mechanical stimulation of the right hind paw of mice previously injected with capsaicin. The antiproliferative properties of these compounds were studied in A427 (nonsmall-cell lung cancer), LCLC-103H (large-cell lung cancer), 5637 (bladder cancer), and DAN-G (pancreatic cancer) cell lines with an *in vitro* crystal violet staining assay. The A427 and 5637 cancer cell lines were the most sensitive to spirocyclic compounds **16**–**20**. Spiropiperidines **20** and **21** were the most potent toward the A427 cell line, with IC_50_ values of 2.6 μM and 5.9 μM, respectively. Considering that the IC_50_ values obtained for the A427 cell line were similar to those obtained with the σ_1_R antagonist haloperidol, the authors assumed that the antiproliferative effect of these compounds was mediated by interference with the activity of σ_1_Rs. In addition, the antiproliferative properties of **20** were partially reversed when the σ_1_R agonist (+)-pentazocine was added. By contrast, results obtained in the bladder cell line (IC_50_ = 5.5 μM and 9.1 μM for **20** and **21**, respectively) seemed to be not related to the interference with σ_1_R. Indeed, (+)-pentazocine exhibited cytotoxic properties on this cell line. In general, compound **20** displayed an unselective cytotoxic effect on all the explored cancer cell lines with the highest value of cytotoxicity (65%) detected for the A427 cell line with the lactate dehydrogenase (LDH) assay. In general, spirocyclic piperidines seemed to act as σ_1_R antagonists with cytotoxic properties. Thus, we suggest that this chemical scaffold may be further exploited by structural simplification or bioisosteric replacements.^114^

#### 3.1.3 Bicyclic Piperazine Derivatives

##### 3.1.3.1 7,9-Diazabicyclo[4.2.2]decane Derivatives

**Figure 12:**
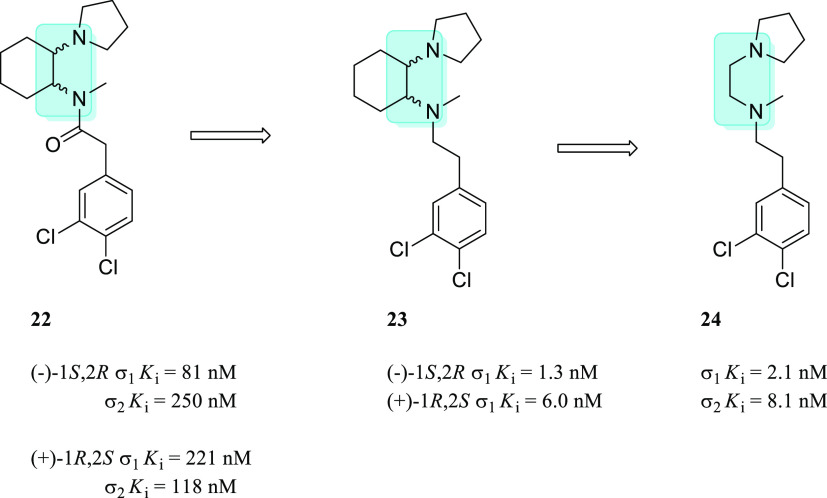
Historically relevant ethylenediamine **22**–**24** with their σRs binding profile. The ethylenediamine structure is highlighted in light blue.

The ethylenediamine moiety has been proven to represent a sufficient chemical substructure that allows a high affinity for the σRs. Indeed, almost 30 years ago, it was discovered that the *cis*-isomers of U50,488 (compound (±)-**22**, Figure 12), a selective KOR agonist, possessed a moderate affinity for the σ_1_R.^115^ Reduction of the amide functional group led to the enantiomeric cyclohexandiamine derivatives of U50,488 (compound (±)-**23**, Figure 12), both containing the ethylenediamine moiety and with high affinity for the σ_1_R.^116^ Removal of the cyclohexane ring afforded the ethylenediamine **24** (Figure 12) with σ_1_*K*_i_ and σ_2_*K*_i_ of 2.1 nM and 8.1 nM, respectively.^117^

**Figure 13:**
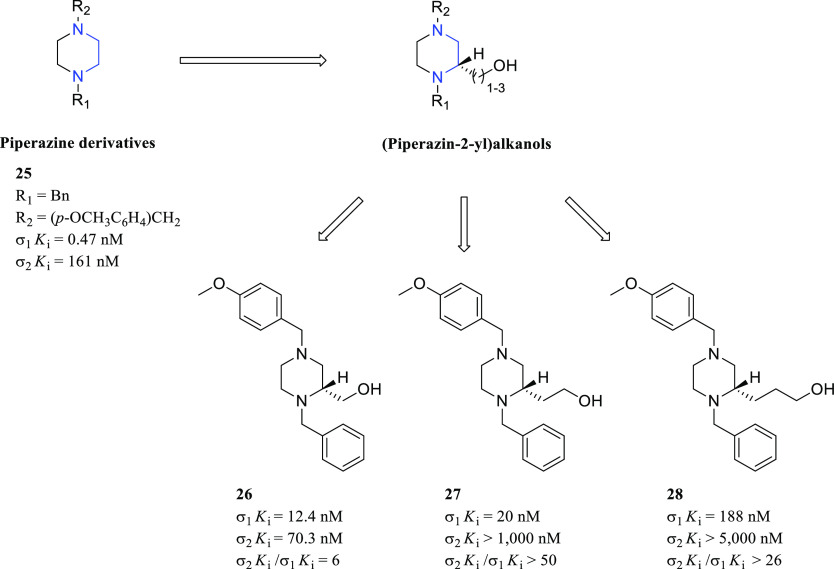
General structures of piperazine derivatives and structures of compounds **25**–**28** with σRs binding profile.

Compound **24** was used as the lead compound for the discovery of novel σR ligands.^118,119^ To expand the SARs of this class of compounds, the ethylenediamine moiety has been included in a conformationally restricted structure, such as the piperazine ring. Specifically, piperazine derivatives were substituted at both nitrogen atoms with hydrophobic substituents to establish a proper binding with the σR protein.^120,121^ The general structure of these compounds is depicted in Figure 13. Among the synthesized compounds, a *p*-methoxybenzyl moiety and a benzyl moiety (compound **25**, Figure 13) led to a σ_1_*K*_i_ value of 0.47 nM. In 2004 and 2012, the discovery of chiral and flexible (piperazin-2-yl)alkanol derivatives with a good affinity toward the σ_1_R was reported (general structure in Figure 13).^122,123^ The best compounds possessed once again a *p*-methoxybenzyl moiety and a benzyl moiety linked to the piperazine nitrogen atoms. The methanol side chain (compound **26**, Figure 13) afforded the best results in terms of σ_1_R affinity (σ_1_*K*_i_ = 12.4 nM), whereas the ethanol side chain (compound **27**, Figure 13) afforded a slightly reduced σ_1_*K*_i_ value (20 nM vs 12.4 nM) but a higher selectivity ratio (σ_2_*K*_i_/σ_1_*K*_i_ > 50). On the other hand, the side chain elongation to three carbon atoms (compound **28**, Figure 13) caused a considerable reduction of affinity (σ_1_*K*_i_ = 188 nM).

**Figure 14:**
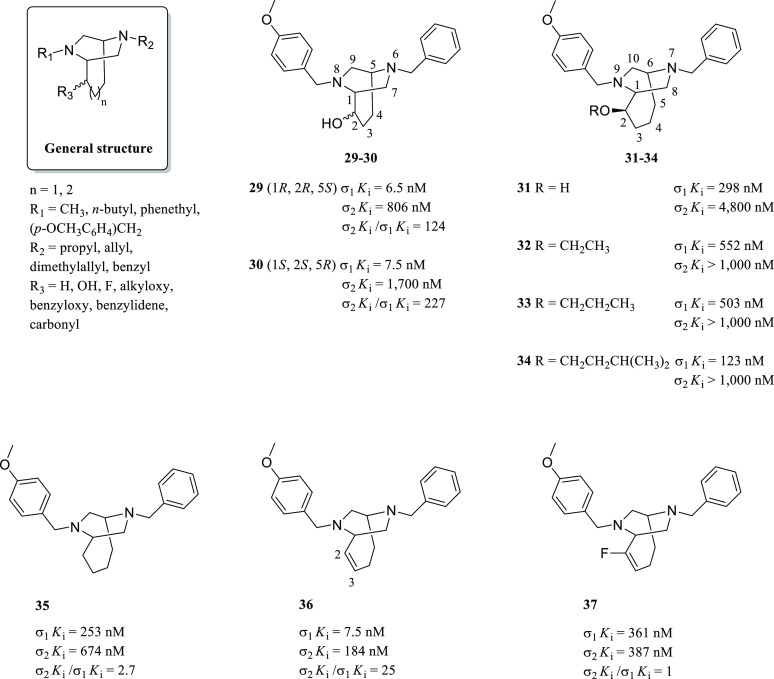
General structure of bicyclic piperazines, chemical structures of 6,8-diazabicyclo[3.2.2]nonanes **29** and **30**, and 7,9-diazabicyclo[4.2.2]decane derivatives **31**–**37** with their σRs binding profile.

With the purpose of defining a 3D pharmacophore model that takes into consideration the appropriate spatial orientation of the pharmacophoric elements of a σ_1_R ligand, a useful strategy is represented by the structural constriction of such elements in a blocked conformation, such as bicyclic structures. With respect to the Glennon pharmacophore model for σ_1_R ligands, the (piperazin-2-yl)alkanol moiety was incorporated in bicyclic frameworks, giving rise to different classes of compounds with a moderate-to-high affinity toward the σ_1_R (general structure in Figure 14).^124−129^ SARs were described for these derivatives. In general, lipophilic substituents at both nitrogen atoms were required for a proper binding with the σ_1_R. The R_2_ group could be an allyl substituent if the carbon atom linked to R_3_ was unsubstituted; otherwise, this unsaturated moiety was not tolerated. In all other cases, R_2_ could be a benzyl, propyl, or dimethylallyl moiety. The R_3_ substituent could be a benzyloxy group if the bridge was made of four carbon atoms. By contrast, if the benzyloxy group was present, a smaller bridge led to a lower affinity. Thus, a benzylidene moiety was allowed. Besides, R_3_ could be a hydroxy group or a carbonyl function only if R_2_ corresponded to a benzyl moiety. Substituents that decreased the basicity of the nitrogen atom linked to R_2_, for example, phenyl or benzoyl, were not tolerated. Finally, bridge annulation with a quinoline or an indole ring or its participation in the formation of spirocycles caused a loss of affinity. In 2007, the synthesis of constrained derivatives of (piperazin-2-yl)propanols possessing a 6,8-diazabicyclo[3.2.2]nonane scaffold and a three-carbon bridge was reported (Figure 14).^130^ Among these derivatives, enantiomeric alcohols **29** and **30** (Figure 14) gave the best results in terms of activity and selectivity. Interestingly, compound **29** was about 30-fold more potent than its corresponding flexible piperazine **28**. The diastereoisomeric alcohols of **29** and **30** (not shown) were about 20-fold less potent (6.5 nM vs 125 nM for compound **29** and its diastereoisomer, 7.5 nM vs 118 nM for compound **30** and its diastereoisomer), suggesting that the orientation of the alcoholic function at the 2-position was somehow crucial to establish a proper interaction with a HBA present in the binding site of the σ_1_R. Also, the authors observed that the alcoholic function of compounds **29** and **30** had a similar spatial orientation of the alcoholic group of compound **26**.

In 2010, Sunnam and co-workers, as a continuation of the work previously described, expanded the carbon bridge of 6,8-diazabicyclo[3.2.2]nonanes from three to four carbon atoms, affording 7,9-diazabicyclo[4.2.2]decane derivatives (compounds **31**–**37**, Figure 14). The authors investigated if ring homologation and modification of the alcoholic function at the 2-position or its complete deletion could affect the activity of this novel class of compounds. Correctly, the homologous compound **29** (alcohol **31**, Figure 14) had only a moderate affinity toward the σ_1_R, whereas the affinity for the σ_2_R fell in the micromolar range. Etherification of the alcoholic function of **31** led to alkyl ethers **32**–**34**. Except for **34**, which possessed a branched isopentyl moiety (σ_1_*K*_i_ = 123 nM), ethers **32** and **33** displayed a worse affinity for the σ_1_R when compared with compound **31**. Elongation of the alkyl chain or insertion of an aromatic ring led to ethers with very low affinity for the σ_1_R (not shown). Also, the authors performed similar structural modifications for the diastereoisomeric alcohols and ethers of **31**–**34**. It was reported that the σ_1_R affinities of such diastereoisomers fell in the micromolar range, emphasizing that in the postulated bioactive conformation, the substituent in position 2 must possess the same spatial orientation observed for compounds **29** and **31**. Removal of the oxygen atom led to alkane **35** with a σ_1_R affinity comparable to **31** (253 nM vs 298 nM, respectively).^131^

Moreover, insertion of a double bond between carbons in positions 2 and 3 on the carbon bridge afforded compound **36**, representing the best compound of this series in terms of σ_1_R affinity. Indeed, compound **36** was about 40-fold more potent than **31** (7.5 nM vs 298 nM, respectively). Unfortunately, compound **36** also displayed the best *K*_i_ value for the σ_2_R for this class of compounds (184 nM). Insertion of a fluorine atom at the 2-position of the double bond led to compound **37** whose σ_1_R affinity was once again comparable with those of compound **31**. SARs were outlined for this class of novel compounds. Comparing the results obtained from the 6,8-diazabicyclo[3.2.2]nonane series and the 7,9-diazabicyclo[4.2.2]decane series, it seemed that homologation of the carbon bridge did not represent a valuable strategy for a better σ_1_R binding. Furthermore, the presence of a substituent at the 2-position of the 7,9-diazabicyclo[4.2.2]decane scaffold was not necessary for the affinity, although stereochemistry represented a factor that must be taken into consideration when such substituents were present. The presence of unsaturation could justify the best result obtained with compound **36**. Indeed, the shorter length of the double bond reduces the bridge size and its flexibility, so that the steric demand of the unsaturated four-carbon bridge and the three-carbon bridge becomes similar with a consequently improved interaction with the σ_1_R (**29**, **30** and **36**). To summarize, enlargement of the bridge size to four atoms in piperazine bicyclic derivatives did not bring a striking beneficial effect for σ_1_R binding unless an unsubstituted double bond was present.

Cytotoxic properties of compounds **35** and **36** were evaluated in a panel of six human tumor cell lines, including A427 (small-cell lung cancer), 5637 (bladder cancer), RT-4 (bladder cancer), LCLC-103H (large-cell lung cancer), MCF-7 (breast cancer), and DAN-G (pancreas cancer). While no cytotoxic activity was observed for RT-4, LCLC-103H, or DAN-G cancer cell lines, good results were obtained mainly with A427 and 5637 cell lines. Specifically, after 96 h of exposure, compound **36** displayed an IC_50_ value of 13 μM for the 5637 cancer cell line and an IC_50_ value of 10 μM for the A427 cancer cell line. Interestingly, the A427 cell line was susceptible to haloperidol, a well-known σ_1_R antagonist, so that the authors hypothesized that compound **36** could act as a σ_1_R antagonist, explaining its antiproliferative activity. However, the same assumption cannot be made for the 5637 cell line, which is insensitive to haloperidol. For these reasons, in our opinion, the assumption of functional activity based on the simple comparison of the biological effect with a reference compound, like haloperidol, is misleading. For this reason, the precise mechanism of cytotoxicity should be investigated more in detail to establish if the cytotoxic properties of compound **36** depend on the selective interaction and inhibition of the σ_1_R.

##### 3.1.3.2 2,5-Diazabicyclo[2.2.2]octane Derivatives

**Figure 15:**
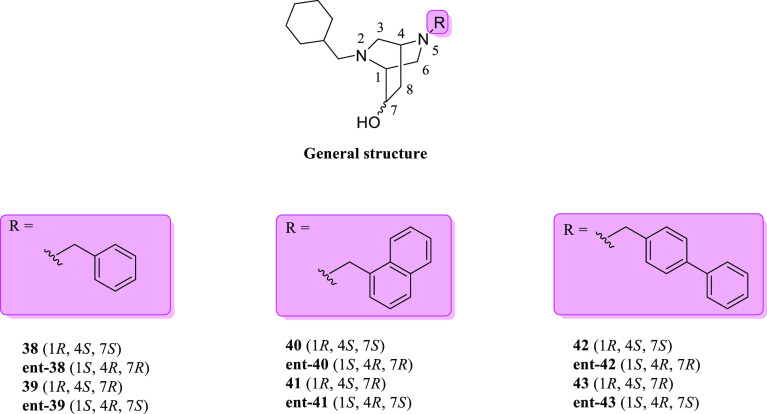
General structure of 2,5-diazabicyclo[2.2.2]octane derivatives **38**–**43** and **ent-38**–**43**.

In 2016, Weber et al. reported the synthesis of 2,5-diazabicyclo[2.2.2]octane derivatives.^132^ These compounds were designed for the same purposes previously discussed for the 7,9-diazabicyclo[4.2.2]decane series. The authors hypothesized that if rigidification of **28** into compound **29** afforded a 30-fold improvement of the σ_1_*K*_i_ affinity value, then similar results should also be achieved by rigidification of the flexible (piperazin-2-yl)-ethanol structure (general structure in Figure 13) into the 2,5-diazabicyclo[2.2.2]octane scaffold (i.e., **38**–**43**, **ent-38**–**43**, Figure 15). The best results in terms of σ_1_R binding (calculated in animal and human myeloma cell lines) were obtained when a cyclohexylmethyl moiety was linked to one nitrogen atom of the bicyclic piperazine (Table 2).

**Table 2:** σ_1_R Binding Profile of Compounds **38**–**43** and **ent-38**–**43**

Compd.	σ_1_*K*_i_ [nM]^a^ (guinea pig brain)	σ_2_*K*_i_ [nM]^a^ (rat liver)	σ_1_*K*_i_ [nM]^a^ (human RPMI 8226 cell line)
**38**	4.8	36	3.2
**ent-38**	23	197	2.8
**39**	6.9	60	2.4
**ent-39**	5.7	501	1.6
**40**	8.0	51	13
**ent-40**	14	40	38
**41**	7.1	157	7.2
**ent-41**	0.50	116	6.0
**42**	8.7	20	27
**ent-42**	11	202	27
**43**	23	334	73
**ent-43**	11	593	24

a Data from ref (132).

Almost all compounds displayed a σ_1_*K*_i_ value <20 nM in guinea pig homogenate, except **ent-38** and **43**. In general, *N*-benzyl derivatives **38**, **ent-38**, **39**, and **ent-39** unveiled better σ_1_*K*_i_ values for the 1-naphtylmethyl and biphenylmethyl derivatives **40**–**43** and **ent-40**–**43** in both animal and human radioligand binding assays (σ_1_*K*_i_ RPMI 8226 cell line <3.0 nM). Interestingly, the 1*S*,4*R*,7*S* configuration ensured the best σ_2_*K*_i_/σ_1_*K*_i_ selectivity ratio (90-fold for **ent-39**, 230-fold for **ent-41**, and 55-fold for **ent-43**). Among all compounds, the naphtylmethyl derivative **ent-41** showed the best affinity toward the σ_1_R (σ_1_*K*_i_ guinea pig brain = 0.50 nM). Generally, stereochemistry seemed to not represent a relevant factor for proper binding to the σ_1_R. Surprisingly, structure rigidification did not significantly improve the σ_1_*K*_i_ values for this class of compounds. Indeed, the authors compared the *K*_i_ values of these novel bicyclic piperazines with those of their parent hydroxyethyl piperazines and saw that they were more or less superimposable.

**Figure 16:**
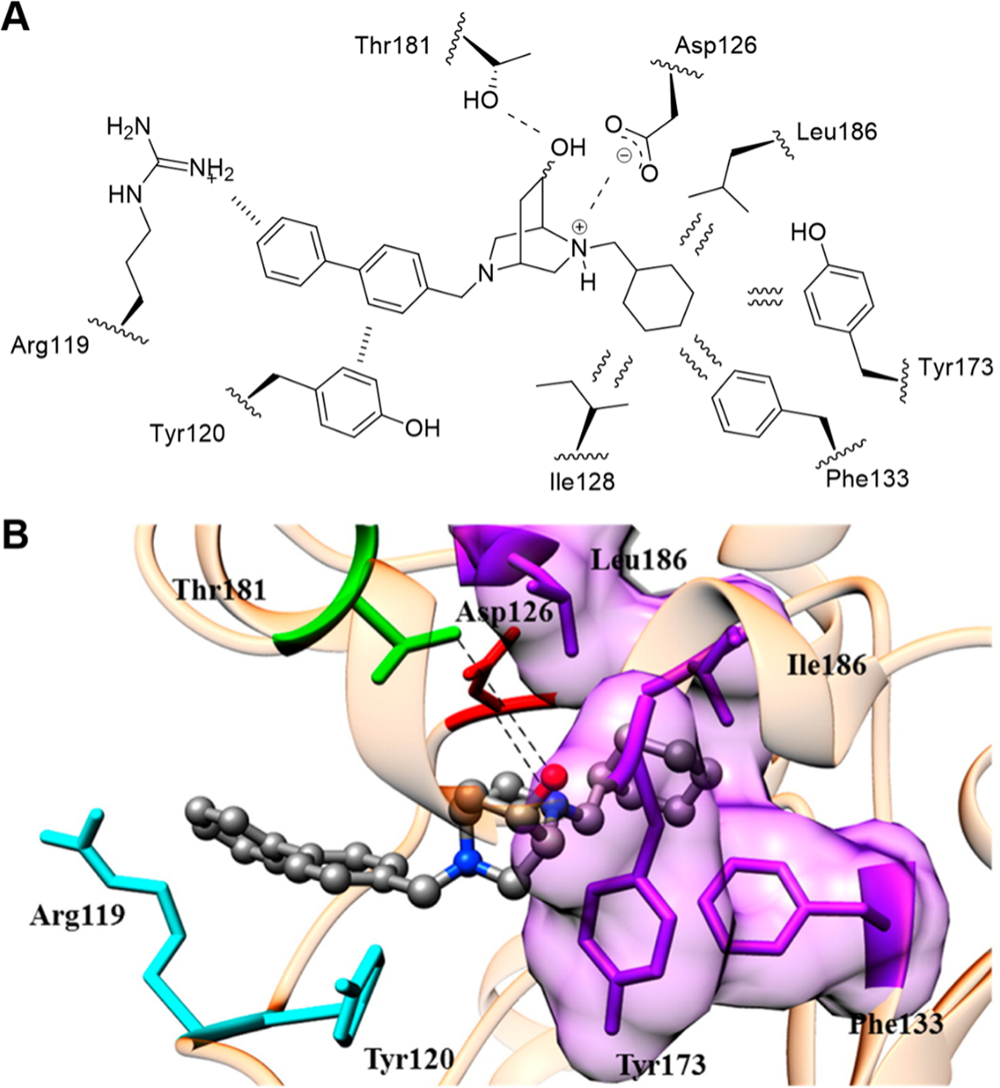
(A) 2D schematic representation of the identified interactions between compound **42** and the main amino acid residues. (B) 3D protein–ligand binding interactions of compound **42** with the σ_1_R homology model. Color-coded as follows: PI (red), HYAr or HYD (light blue), HY (pink), HBA (light green), π-interactions (Arg119 and Tyr120, cyan), salt bridge (Asp126, red), hydrophobic interactions (Ile128, Phe133, Tyr173, and Leu186, purple), and hydrogen bond (Thr181, green). Adapted with permission from ref (132). Copyright 2016 American Chemical Society.

Molecular modeling studies were performed in order to explain these unexpected results. For both classes of compounds, binding free energy values were calculated and compared. Results showed that rigidification of the flexible hydroxyethyl piperazine structure into the 2,5-diazabicyclo[2.2.2]octane scaffold determined a slightly favorable increase of the entropic binding component value. The enthalpic–entropic compensation observed for the 2,5-diazabicyclo[2.2.2]octane class of compounds determined binding free energy values perfectly comparable with the binding free energy values calculated for their parent hydroxyethyl piperazines. In addition, these studies also highlighted the binding determinants of bicyclic piperazine derivatives as follows: (i) the cyclohexyl methyl moiety is buried in a hydrophobic pocket of the receptor; (ii) the aromatic portion of the molecule is involved in the formation of π–π and π-cation interactions; (iii) the nitrogen atom linked to the aryl methyl moiety establishes a salt bridge bond with the carboxylic residue of Asp126; and (iv) the hydroxy group assumes the role of a HBA (Figure 16).

Cytotoxicity studies on 2,5-diazabicyclo[2.2.2]octanes **38**–**43** and **ent-38**–**43** were performed using the crystal violet assay in seven cancer cell lines: 5637 and RT-4 (bladder cancer), DAN-G (pancreatic cancer), MCF-7 (breast cancer), A427 (small-cell lung cancer), and LCLC-103H (large-cell lung cancer). Compounds **40** and **41** displayed unselective growth inhibition, whereas the 5637 cell line was slightly sensitive to compounds **ent-38**, **39**, **ent-41**, and **42**. Except for unselective cytotoxic compounds **40** and **41**, the other bicyclic piperazines exhibited potent IC_50_ values ranging from 1.6 to 4.3 μM for the A427 cell line. The higher susceptibility of A427 cells can be attributed to their overexpression of σ_1_Rs,^130^ as previously stated for spiropiperidines **16**–**21**. In depth studies on double-stained A427 cells with annexin V-FITC and propidium iodide were made to investigate apoptosis induction by compounds **ent-38** and **ent-40**–**42**. After 24 h, the biphenylmethyl compound **ent-42** caused the appearance of about 40% of early apoptotic cells. On the other hand, all other tested compounds induced apoptosis only after 48 h. Considering that the induced growth inhibition effect mediated by these compounds on A427 cells was similar to that of haloperidol, the authors assumed that 2,5-diazabicyclo[2.2.2]octanes acted as σ_1_R antagonists. However, we would like to stress that as a good practice in defining the putative functional role of new σR ligands, additional *in vivo* studies (e.g., formalin mice assay) are needed to validate such generalizations.

### 3.2 Selective σ_2_R Ligands

**Figure 17:**
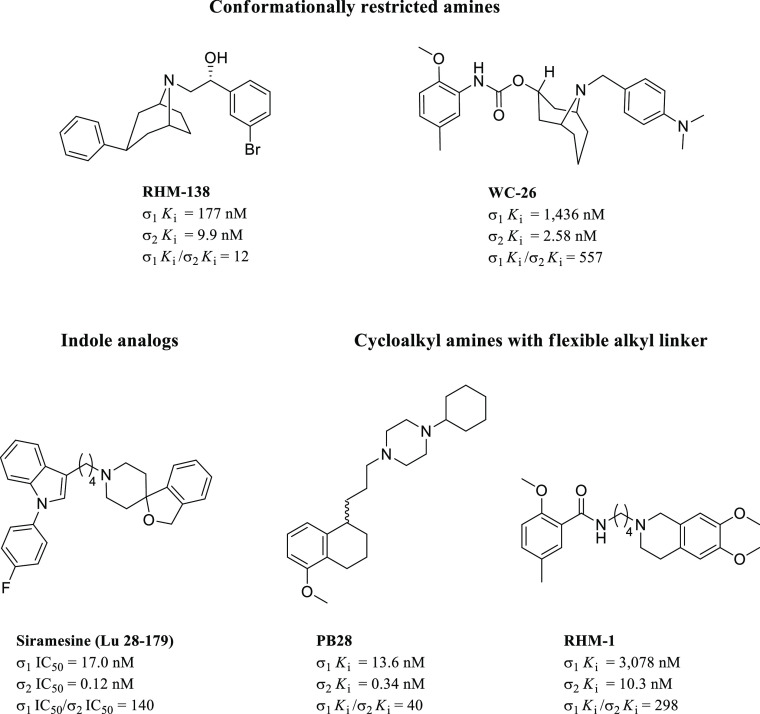
Representative structures for different chemical classes of σ_2_R ligands.

To date, the development of truly selective σ_2_R ligands has been challenging due to the vast and heterogeneous range of structures that can fit into the σ_2_R binding site. These chemical classes include conformationally restricted amines (e.g., benzomorphan-7-one, granatane, and methanobenzazocine derivatives), indole analogs compounds (e.g., siramesine-related derivatives),^133^ and cycloalkyl amines with a flexible alkyl linker (e.g., *N*-cyclohexylpiperazine, *N*-(4-fluorophenyl)piperazine, and 6,7-dimethoxy-1,2,3,4-tetrahydroisoquinoline derivatives).^134−136^ A few examples of such representative structures are depicted in Figure 17. Concerning their cytotoxicity properties, siramesine showed to induce cell death through a p53- and caspase-independent apoptotic pathway.^72^ On the other hand, the dose-dependent effect exerted by the tropane derivative RHM-138 was mediated by caspase-dependent apoptosis.^137^ The highly selective granatane derivative WC-26 and the cyclohexylpiperazine derivative PB28 enhanced the cytotoxicity of existing anticancer drugs, such as doxorubicin, by increasing the intracellular reactive oxygen species (ROS) or decreasing the expression of the P-glycoprotein (P-gp), respectively.^138,139^ Finally, benzamide derivative RHM-1 did not induce cytotoxicity and caspase-3 activation. However, due to the favorable binding profile, it was radiolabeled and further developed as a PET tracer for cancer diagnosis.^140^

#### 3.2.1 Conformationally Restricted Amines: Selective Granatane Derivatives

**Figure 18:**
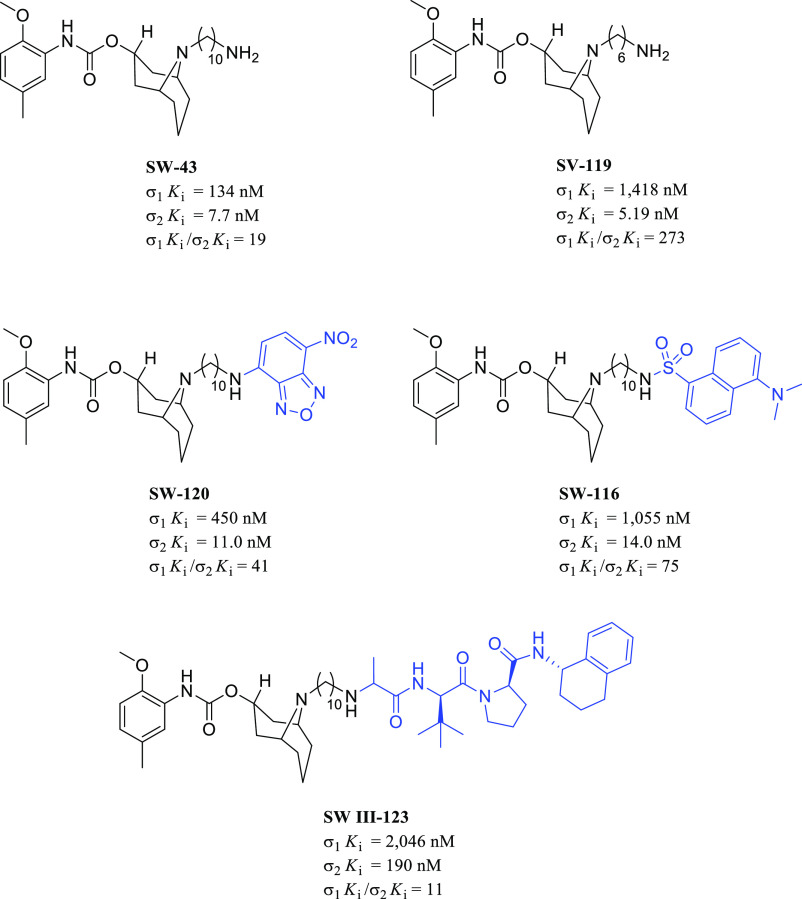
Chemical structure and σRs binding profile of selective *N*-substituted 9-azabicyclo[3.3.1]nonan-3α-yl phenylcarbamate derivatives and conjugated derivative SW III-123.

The σRs’ ability to bind tropane-based molecules, including cocaine,^141^ drove much of the early interest in the development of this class and structurally related compounds such as granatane derivatives.^138,142,143^ In 2010, Hornick et al. evaluated the capability of a novel granatane-based σ_2_R ligand to induce apoptosis and augment standard chemotherapy in pancreas cancer.^144^ SW-43, bearing a 9-azabicyclo[3.3.1]nonan-3α-yl ring with an aminoalkyl extension, showed a higher effect on tumor cell viability when compared to the structural related analog SV-119 (Figure 18), even though a loss of σ_2_R affinity and selectivity occurred. Indeed, shortening the length of the aminoalkyl chain from 10 (SW-43) to 6 (SV-119) carbons increased the σ_1_*K*_i_/σ_2_*K*_i_ selectivity ratio significantly (19 vs 273).^143^ However, the higher lipophilicity of SW-43 might have helped to enhance the membrane diffusion into the cell.^144^ Moreover, the *in vivo* antitumor effects of the commercially available siramesine were also compared with that of the two granatane-based compounds. Thus, σ_2_R ligands treatment decreased tumor volume to the same extent as gemcitabine, while the combination of compound SW-43 with gemcitabine resulted in a superior effect in the stabilization of tumor volume than other tested compounds.^144^

The primary amine function of compound SW-43 was successively condensed with 4-chloro-7-nitrobenzo-2-oxa-1,3-diazole or 5-(dimethylamino)naphthalene-1-sulfonyl chloride (Figure 18), which acted as fluorophores, to develop novel fluorescent σ_2_R selective ligands SW-120 and SW-116 for imaging of cell proliferation.^145^ SAR analysis on granatane analogs suggested that a broad range of *N*-substitutions of the 9-azabicyclo[3.3.1]nonane was highly tolerated. Indeed, the introduction of extraordinarily long and large substituents (e.g., ω-amino groups and substituted benzo-fused heterocycles) did not affect σ_2_R affinity and selectivity significantly (WC-26 vs SV-119), while the *N*-substitution bearing an additional nitrogen atom located at least five carbon units apart led to increased affinity for the σ_2_R. Finally, the presence of aryl groups on the *N*-substituent was not essential for both affinity and selectivity for the σ_2_R (SW-43), although it was permitted.

In a follow-up study, the structure of SW-43 was conjugated with that of a second mitochondria-derived activator of caspase (SMAC) compound to develop an innovative class of tumor-targeting drug delivery agents for treating ovarian cancer.^146^ As a result, the new hybrid compound named SW III-123 (Figure 18) retained a sufficient σ_2_R affinity to allow the successful delivery of the SMAC compound into ovarian cancer cells. The finding was supported by the potent cytotoxic effect of the new compound toward different human ovarian cancer cell lines (i.e., SKOV-3, CaOV-3, and BG-1) after 24 h treatment, which was not due to synergistic effects of the two molecules since their combination produced less cytotoxicity than the conjugated compound.^146^ The strategy proposed by Zeng et al. was an interesting attempt; however, a significant limitation is the absence of either improved cytotoxic effect or synergism between the simultaneous modulation of the two targets. From our perspective, a different conjugation strategy (e.g., not cleavable vs cleavable linker) of the two active small molecules might be beneficial to overcome this issue. Therefore, as a future investigation, we suggest applying the mutual prodrugs approach to develop novel conjugates with bivalent function, that is, to obtain the synergistic effect and develop an effective drug delivery system. Consequently, by using selective σ_2_R ligands as a suitable promoiety, it might be possible to take advantage of both its antiproliferative effect (active promoiety) and tumor-targeting drug delivery properties (carrier promoiety).

#### 3.2.2 Siramesine-Related Compounds: Selective Indole Derivatives

Siramesine (Figure 17) has been reported, by Perregaard et al. in 1995, as a first selective σ_2_R ligand with relatively low affinity for additional off-targets, including 5-HT_1A_R, 5-HT_2A_R, D_2_R, and α_1_R.^133^ Although siramesine was initially developed as a nontoxic CNS agent with potent anxiolytic activity,^147−150^ later, it has been extensively evaluated both *in vitro* and *in vivo* for its antitumor properties and used as a reference σ_2_R agonist accordingly.^151−155^ Nevertheless, it has been demonstrated that siramesine-mediated cell death was likely due to the modulation of multiple molecular targets rather than through exclusively σ_2_Rs activation.^156^ Precisely, siramesine seemed to act as a lysosomotropic agent able to impact lysosomal membrane permeabilization and leakage, leading to increased ROS and triggering apoptosis signaling and cell death.^72,152^ More recently, the combination of siramesine and lapatinib (a dual tyrosine kinase inhibitor) was reported to induce cell death in MDA MB-231 and SKBR3 breast cancer cell lines mediating ferroptosis and autophagy through an unclear synergistic effect.^157^

**Figure 19:**
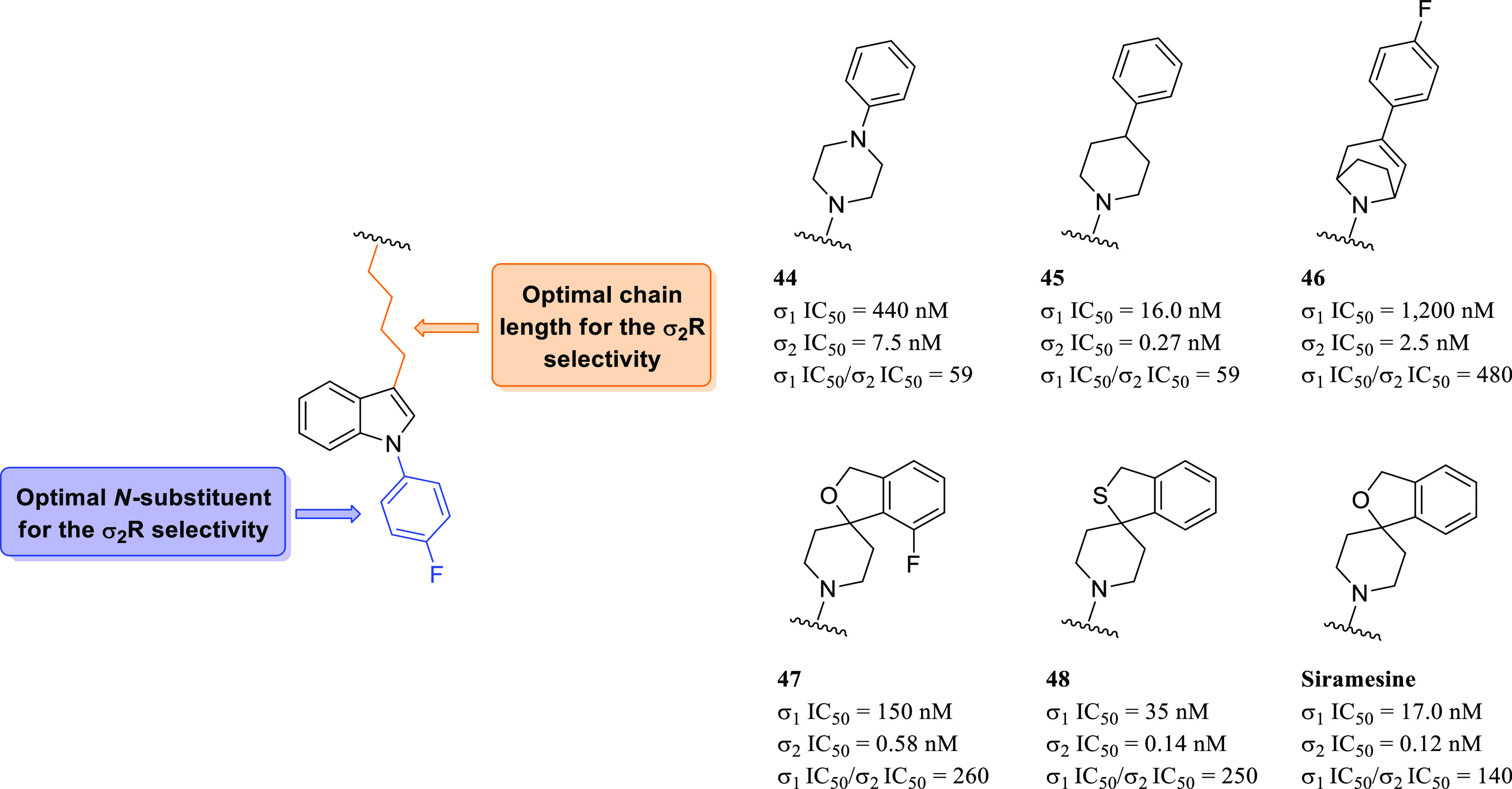
Early structural modification of siramesine and its analogs **44**–**48**.

Since its discovery, the chemical structure of siramesine has been manipulated to obtain improved highly selective indole analogs (Figure 19). Mainly, modification of both the indole scaffold and the spiropiperidine moiety was carried out; thus, structural determinants for this class of σRs ligands were extensively explored.^109,110,158,159^

Earlier SAR studies on indole analogs revealed that a concurrent presence of a butyl chain as a spacer and a 4-fluorophenyl substituent at the indole ring increased the σ_2_R selectivity considerably.^133^ On the other hand, the arylpiperidine moiety induced higher σRs affinity than arylpiperazine (**45** vs **44**), while their replacement with a spiro[isobenzofuran-l(3*H*),4′-piperidine] resulted in a more selective compound (siramesine vs **44** and **45**). Concerning the σ_1_*K*_i_/σ_2_*K*_i_ selectivity ratio, the best result was obtained by the tropane derivative **46**.

A subsequent study aimed to determine the structural elements leading the σRs affinity and selectivity within the indole analogs class was performed by synthesizing spiro-joined benzofuran, isobenzofuran, and benzopyran piperidine derivatives.^109^ Accordingly, two major critical features were found: (i) larger lipophilic *N*-substituents at the spiro-joined isobenzofuran ring promoted the σ_2_R affinity (H < Me < Et < *i*-Pr < *n*-Pr < *n*-Bu < (CH_2_)_4_Ph); and (ii) a substituent at the benzene ring of the spiropiperidine system greatly affected the σ_1_R/σ_2_R ratio (*i*-Pr < Me < 4-CF_3_ < 4-*F* < 7-F), as exemplified by **47**, while changing in the geometry of the spiro-system (e.g., benzofuran and benzopyran) decreased the σ_2_R affinity. Finally, exchanging the isobenzofuran portion of siramesine with the thioisobenzofuran moiety further increased the σ_2_R selectivity (siramesine vs **48**).

**Figure 20:**
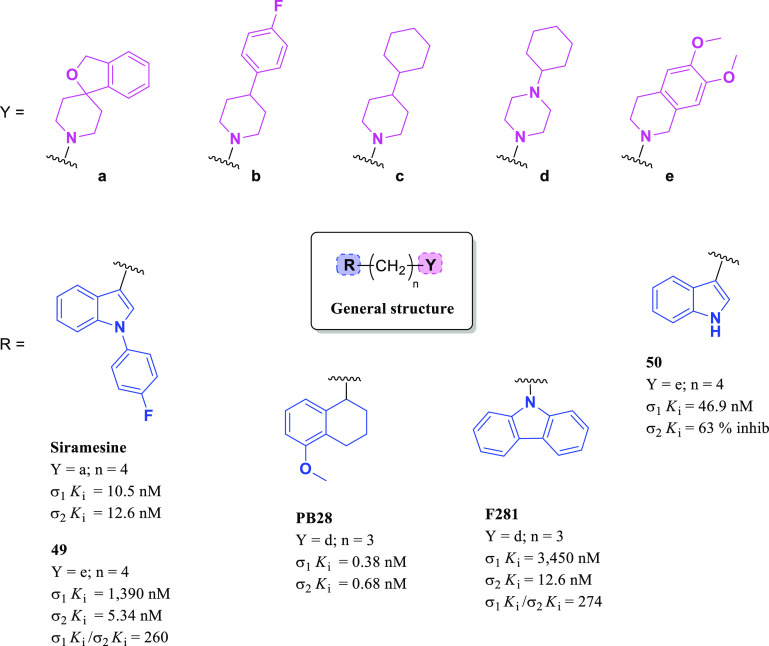
General structure and σRs binding profile of siramesine-related derivatives.

Niso et al. described the development of novel σ_2_R agonists as possible antitumor agents in multidrug-resistant cancers.^158^ The newly synthesized compounds possessed different and heterogeneous scaffolds, such as 1-(4-fluorophenyl)-1*H*-indole, 1*H*-indole, 5-methoxy-1,2,3,4-tetrahydronaphthalene, and 9*H*-carbazole, which were selected based on the structure of different reference compounds, for instance, siramesine, PB28, and F281 (Figure 20). Also, to combine the structural features probably responsible for high σ_2_R affinity, specific cyclic amine moieties (Figure 20) were alternatively connected to the scaffolds forming four different series. Among the indole series, the *N*-substituted analogs were more selective for the σ_2_R than *N*-unsubstituted ones (**49** vs **50**). Thus, the authors suggested that the preferred σ_1_R affinity observed for indole analogs might be due to an additional hydrogen bond formed between the NH group belonging to the indole and the σ_1_R. These data were consistent with that previously reported by Perregaard et al. On the other hand, the σ_1_R affinity value of siramesine (*K*_i_ = 10.5 nM, Figure 20) was found to be much higher than discovered initially, causing a tremendous reduction of the σ_1_*K*_i_/σ_2_*K*_i_ selectivity ratio.^158^ For the sake of clarity, these inconsistent data are indeed most likely due to the slightly different binding protocols adopted. Similarly, PB28, originally described as a high-preferred σ_2_R agonist (σ_1_*K*_i_/σ_2_*K*_i_ = 40), was found to possess a more significant affinity for the σ_1_R (σ_1_*K*_i_ = 0.38 nM and σ_2_*K*_i_ = 0.68 nM). Despite the σ_1_R/σ_2_R mixed profile of PB28, this cyclohexylpiperazine derivative emerged as one of the most potent putative σ_1_R antagonist/σ_2_R agonist known until today, and as we will discuss in the next section, it has been extensively studied both for its biological activity and the SAfiRs as a lead compound.^160^ Interestingly, PB28 has been recently tested for its *in vitro* anti-SARS-CoV-2 activity, and it was found to be more potent and less cardiotoxic than hydroxychloroquine, supporting further studies as a promising pan-viral candidate.^161^

Compound **49** (Figure 20), a siramesine analog, displayed notable σ_2_R selectivity over the σ_1_R subtype as well as significant antiproliferative activity in human breast cancer cells, either sensitive or resistant to doxorubicin (EC_50_ = 17.8 and 21.8 μM, in MCF-7 and MCF-7/dox, respectively). Furthermore, **49** interacted with P-gp stronger than siramesine (EC_50_ = 0.21 and 1.41 μM, respectively) and restored the antitumor activity of doxorubicin after co-administration with it, suggesting efficacy in cells with P-gp-induced resistance.^158^

**Figure 21:**
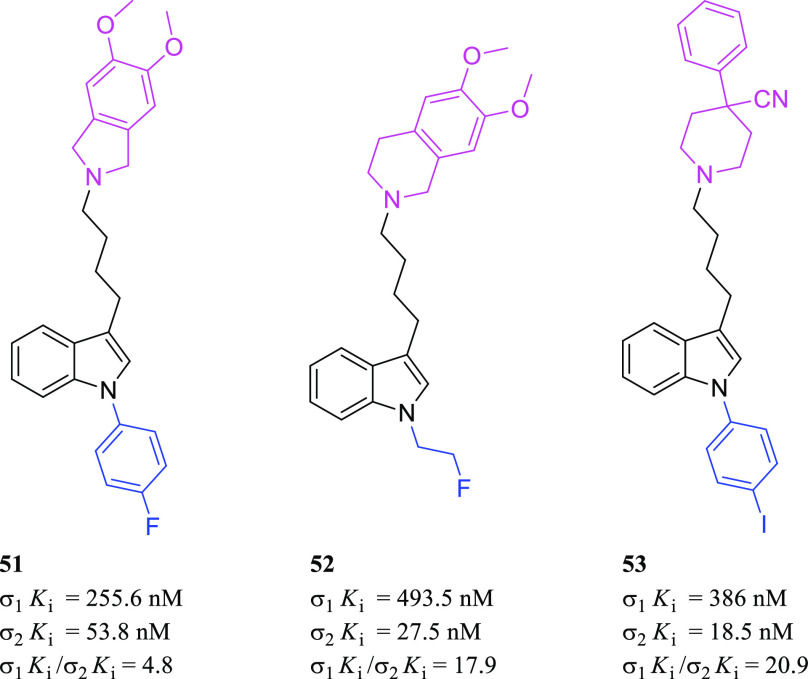
General structure and σRs binding profile of siramesine-related derivatives **51**, **52**, and **53**.

In 2015, Xie et al. reported the synthesis, SAfiRs, and antiproliferative activity of a series of indole-based σ_2_R ligands derived from siramesine.^159^ To develop new σ_2_R ligands and find valuable radiotracers for tumor imaging, the authors applied three different modifications to the siramesine’s structure (**51**–**53**, Figure 21). Notably, both the spiro-joined isobenzofuran and the indole *N*-substitution regions of siramesine were explored by replacing them with different preferred σRs cyclic amines, including 5,6-dimethoxyisoindoline (**51**), 6,7-dimethoxy-1,2,3,4-tetrahydroisoquinoline (**52**), 4-phenylpiperidine-4-carbonitrile (**53**), and different fluoroalkoxy-phenyl-piperazines (not shown), or with a 2-fluoroalkyl group (**52**) and a *N*-(4-iodophenyl) group (**53**), respectively. Subsequently, both portions were concurrently modified, like in **53**. SARs performed on this series were consistent with previously reported studies, confirming the critical role of both the σ_2_R-preferred cyclic amine motif and the *N*-(4-fluorophenyl)indole scaffold to increase the σ_2_R affinity and selectivity. On the other hand, a consistent discrepancy in the σ_2_*K*i values with those reported by Niso et al. was observed for compound **49** (σ_1_*K*_i_ = 530.8 nM and σ_2_*K*_i_ = 49.2 nM vs σ_1_*K*_i_ = 1,390 nM and σ_2_*K*_i_ = 5.34 nM) which resulted in a substantial loss of subtype selectivity (σ_1_*K*_i_/σ_2_*K*_i_ = 260 vs 11). Nevertheless, compounds **49** and its 5,6-dimethoxyisoindolineanalog (**51**) (σ_1_*K*_i_ = 255.6 nM and σ_2_*K*_i_ = 53.8 nM) were tested in the 3-(4,5-dimethylthiazol-2-yl)-2,5-diphenyltetrazolium bromide (MTT) assay to evaluate their antiproliferative activity in DU145, MCF-7, and C6 cancer cells along with siramesine used as a reference compound. Both compounds showed EC_50_ values comparable to that of siramesine in all the tested cell lines, with the highest antiproliferative activity exerted by **51** in MCF-7 cells (EC_50_ = 17.0 μM). Moreover, cell cycle analysis using flow cytometry revealed that **49**, **51**, and siramesine induced G_1_ phase cell cycle arrest in DU145 cells.

Very recently, compound **49**, with other two low-affinity σ_2_R ligands (not shown), was reported as the first-in-class multidrug resistance-associated protein 1 (MRP1) modulator acting as a collateral sensitizer.^162^ Higher cytotoxicity effects were observed in the MRP1 overexpressing cells (i.e., MDCK/MRP1 and A549/DX) than in the wild-type counterparts, supporting the involvement of the collateral sensitivity-mediated activity. Furthermore, co-administration of **49** with cisplatin in a A549/DX xenografts model showed a significant reduction in tumor growth, while the single-agent administration did not.^162^

#### 3.2.3 Cycloalkyl Amines with Flexible Alkyl Linker: Substituted Piperazine/Piperidine and Tetrahydroisoquinoline Derivatives

##### 3.2.3.1 Cyclohexylpiperazine and Cyclohexylpiperidine Analogs

**Figure 22:**
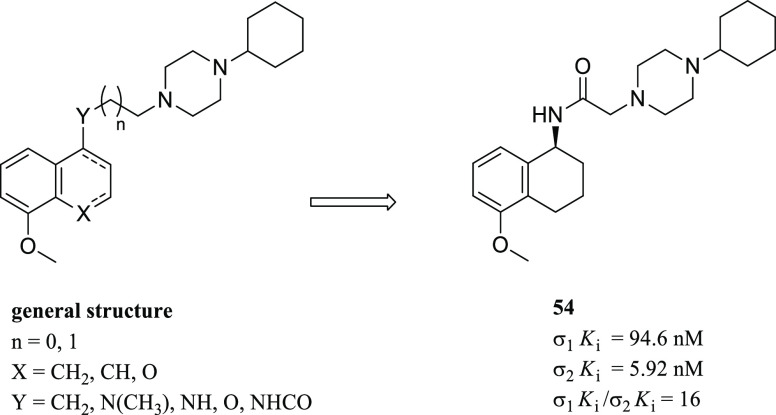
General structure of PB28 analogs with reduced lipophilicity and σRs binding profile of compound **54**.

Cyclohexylpiperazine derivatives represent a broad set of well-studied σRs ligands, with PB28 (Figure 17) being the prototype compound for this class. This tetralin-based σR-preferred ligand has been extensively investigated for its anticancer properties,^36,41,163^ and many PB28 related analogs were prepared over the past years.^164^ Particularly, specific modifications of the PB28 structure, aiming to obtain optimal log *P* and log *D* values to reduce nonspecific binding and improve cancer cells intake of new analogs, were performed.^103^ With this in mind, in 2011, Abate et al. synthesized new PB28 analogs with reduced lipophilicity by introducing a polar functional group (i.e., amine, amide, or ether group) in the propylene linker or by replacing the tetralin with a chromane nucleus (Figure 22). In addition, pure enantiomers were obtained whenever possible, and a naphthalene ring instead of the tetralin one was used to evaluate the effect of the chirality on the σ_2_R affinity. Unfortunately, none of the newly less lipophilic analogs showed better affinity or selectivity than PB28. Compound **54** (Figure 22) displayed the best binding profile through the series and suitable lipophilicity to enter tumor cells. However, **54** did not exert antiproliferative activity in SK-N-SH cells, while it showed specific activity toward the P-gp efflux pump (EC_50_ = 8.1 μM), suggesting a few limitations in its further development as a diagnostic or therapeutic agent.

**Figure 23:**
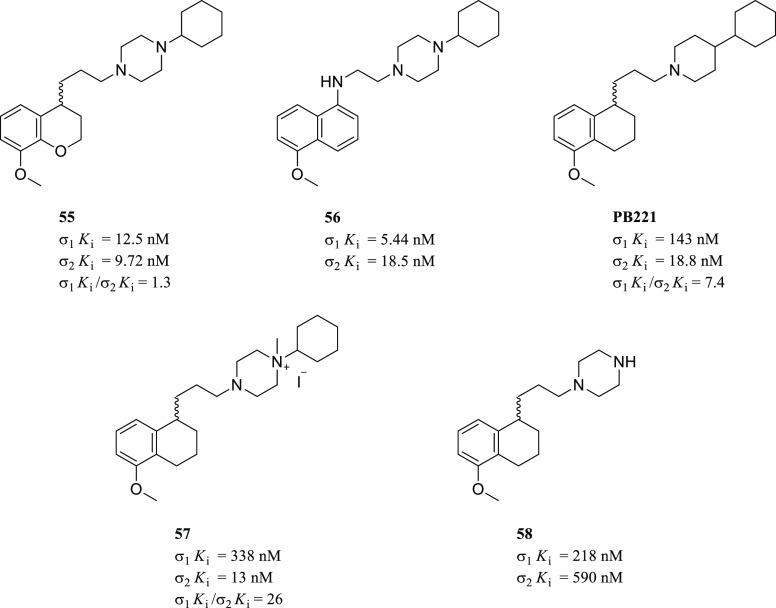
Representative structural modification for PB28 analogs on propylene linker and tetralin scaffold (**55** and **56**), piperazine ring (PB221), basic *N*-atom (**57**), piperazine substitution (**58**).

Interestingly, these results, along with previously reported ones from the same authors,^165^ supported the fact that lipophilicity played a pivotal role in the σ_2_R activity. In contrast, the enantioselectivity had only a marginal effect on receptor subtypes interactions. Based on σRs binding data and the extensive modifications performed on PB28 structure (**55**–**58** and PB221, Figure 23), the following SAfiRs can be summarized: (i) introduction of a polar functional group either in the propylene linker or in the tetralin scaffold of PB28 reduced the σ_2_R affinity (PB28 vs **55** and **56**); (ii) piperazine ring replacement or opening led to decrease of the σRs affinity (PB28 vs PB221); (iii) modification of the *N*-atom connected to the cyclohexyl group (e.g., substitution, quaternization or incorporation into an amide function) mainly affected the affinity at the σ_1_R subtype (PB28 vs **57**); however, the presence of both basic *N*-atoms is needed for higher σ_2_R affinity; and (iv) a cyclohexyl group as a substituent at the piperazine ring is optimal for the σRs affinity (PB28 vs **58**).

The preclinical efficacy toward pancreatic tumor models of PB28-related compounds, including F281 and PB221, was investigated by Pati et al.^36^ The cytotoxic effect after 24 h exposure to the tested compounds was assessed on different human and mouse pancreatic cancer cell lines (i.e., MIAPaCa-2, BxPC3, AsPC-1, Panc-1 and Panc02, KP-2, and KCKO, respectively). Heterogeneous outcomes on cell viability were observed in a cancer cell lines manner. For example, the cytotoxic effect was more significant for specific cell lines such as Panc02, while AsPC1 and Panc-1 resulted in the most resistance among the selected cell lines. Among the tested ligands, both F281 and PB221 displayed the best *in vitro* antiproliferative profile toward the cells panel. A significant increase in caspase-3 *in vitro* activity was detected for PB221, supporting the caspase-dependent apoptotic pathway mediated by its σ_2_R activity. Also, a substantial increase in mitochondrial superoxide radical production was observed. On the other hand, generally, a poor match between *in vitro* and *in vivo* efficacy occurred, except for daily treatment with PB28, which produced a similar *in vivo* effect to that of gemcitabine alone. To justify these results, the authors suggested the formation of active metabolites for the most potent compounds. However, no metabolic stability studies were performed to support this speculation.

Very recently, the antitumor effect of the 4-cyclohexylpiperidine derivative PB221 on an anaplastic astrocytoma tumor model has been explored.^79^ To pursue this goal, both the murine brain tumor cell line ALTS1C1 and the murine pancreatic cell line UN-KC6141 were initially used to examine the compound’s cytotoxic properties. The IC_50_ values of PB221 were found to be 10.61 μM and 13.13 μM for ALTS1C1 and UN-KC6141 cell lines, respectively. However, α-tocopherol (but not *N*-acetylcysteine) counteracted these effects, suggesting the involvement of mitochondrial superoxide production.^79^ Besides, *in vivo* studies performed on C57BL/6 J mice showed that PB221 delayed tumor growth up to 36% compared to the control and increased the survival time from 26 to 31 days in the orthotopic tumor model. Interestingly, PB221 was well tolerated at the tested dose (1 mg/mouse/injection), showing similar side effects to the approved drug Temozolomide.

##### 3.2.3.2 *N*-(4-Fluorophenyl)piperazine Analogs

**Figure 24:**
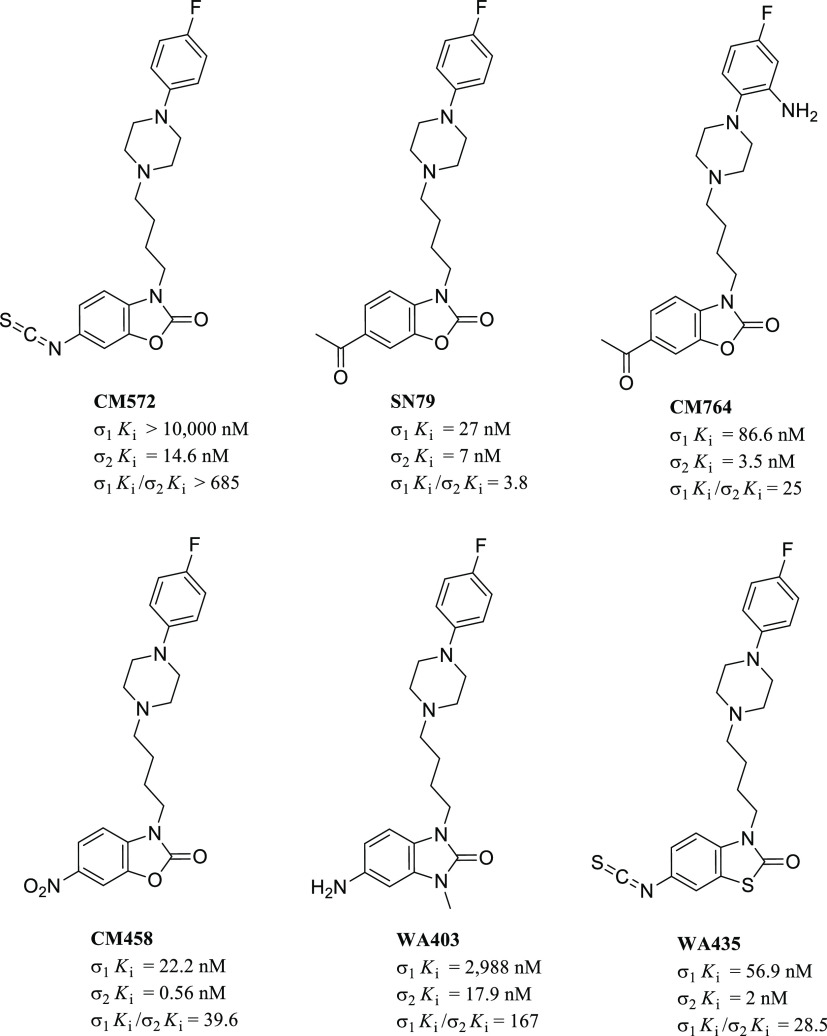
Chemical structure and σRs binding profile of *N*-(4-fluorophenyl)piperazine derivatives.

McCurdy and co-workers carried out extensive research on developing selective σ_2_R probes to elucidate the receptor’s functional roles in several medical conditions, including cancer.^51,55,138^^112,166,167^ Notably, in 2015, Nicholson et al. reported the pharmacological characterization of a σ_2_R-preferred ligand bearing the *N*-(4-fluorophenyl)piperazine as a cyclic amine moiety (CM572, Figure 24).^166^ This new compound was initially developed within a set of isothiocyanate analogs of SN79 (Figure 24), a well-characterized mixed σ_1_R/σ_2_R antagonist (σ_1_*K*_i_ = 27 nM, and σ_2_*K*_i_ = 7 nM). To obtain irreversible σ_2_R binding, the authors incorporated the isothiocyanate group at the 6-position of the 1,3-benzoxazol-2(3*H*)-one scaffold. Furthermore, the introduction of the 6-isothiocyanate moiety (CM572) instead of the 6-acetyl group (SN79) was detrimental for the σ_1_R binding, with a consequent increase of the σ_2_R selectivity (σ_1_*K*_i_/σ_2_*K*_i_ = 685, Figure 24). Interestingly, CM572 showed a dose-dependent calcium response in a neuroblastoma cancer cell line (SK-N-SH) at higher doses, supporting its partial agonist properties at the σ_2_R. Subsequently, the cytotoxicity of CM572 was evaluated against three different cancer cell lines (i.e., SK-N-SH, PANC-1, and MCF-7) as well as toward normal cells such as primary epidermal melanocytes and human mammary epithelial cells. As a result, the cytotoxic effect for CM572 was higher in cancer cells than normal cells, significantly CM572 showed to induce dose-dependent cell death (EC_50_ = 7.6 μM) after 24 h treatment of SK-N-SH cells.^166^

The same research team investigated the non-apoptotic and stimulatory effects on glycolytic cellular metabolism exerted by some of their σ_2_R selective ligands. In particular, based on the pharmacological characterization of compound CM764, a new metabolic regulatory function for σ_2_R was proposed. The novel benzoxazolone analog of SN79, which differed from the parent compound only in the amino group at 2-position of the 4-fluorophenylpiperazine moiety (Figure 24), was initially assessed in a radioligand binding competition assay, revealing 25-fold selectivity over the σ_1_R with an improvement in the σ_2_R affinity (CM764 vs SN79). Interestingly, CM764 increased the MTT reduction in SK-N-SH neuroblastoma cells without inducing changes in cell viability or cell proliferation. In addition, the increase in MTT reduction was partially or entirely blocked by different σ_2_R antagonists, suggesting a σ_2_R-mediated mechanism. Moreover, the overall stimulatory effect included an increased level of NAD^+^/NADH and ATP, a reduction in ROS, and an increment of both the hypoxia-inducible factor 1α and the vascular endothelial growth factor levels. Altogether, the data suggested that σ_2_R ligands with different functional profiles could modulate dual cellular pathways (death vs survival).^112^

In a more recent study, the divergent cytotoxic and metabolically stimulative effects of *N*-(4-fluorophenyl)piperazines were further examined.^167^ Also, the structural determinants required to design selective σ_2_R with predicted dual functions were analyzed. The tested series encompassed σR ligands structurally related to compounds CM572 and SN79 (included), where single-element variations at the 6-position of the 1,3-benzoxazol-2(3*H*)-one, 3-methyl-1*H*-benzimidazol-2-one, and 1,3-benzothiazol-2(3*H*)-one heterocyclic systems were applied (CM458, WA403, and WA435, Figure 24). Compound CM458 bearing a nitro functional group at the 6-position of the benzoxazolone ring stood out for its subnanomolar affinity at the σ_2_R (*K*_i_ = 0.56 nM), while the 5-amino-3-methyl-benzimidazolone analog WA403 showed the best selectivity ratio (σ_1_*K*_i_/σ_2_*K*_i_ = 167) among the series. Generally, an isothiocyanate group as a substituent reduced the σ_1_R affinity, thus increasing the σ_2_R selectivity (CM572 and WA435 vs SN79, Figure 24). However, for the benzothiazolone analog WA435, the loss of σ_1_R affinity was less remarkable. Notably, the new SN79 analogs were at least 25-fold more selective for the σ_2_R than the parent compound. Concerning the divergent effects elicited by *N*-(4-fluorophenyl)piperazine analogs, the following SARs were found: (i) introduction of the 6-isothiocyanate group, regardless of heterocycle, potently induced programmed cell death most likely due to the irreversible receptor binding; (ii) substitution at the 6-position with acetyl, nitro, amino, or fluorine did not produce a significant cytotoxic effect; therefore, the presence of a highly electron-withdrawing group is not sufficient to obtain cytotoxicity; and (iii) changing in the heterocycle system was not decisive for the divergent effect. Finally, other non-isothiocyanate derivatives, including SN79, possibly acting as putative σ_2_R antagonists, were tagged as “neutral” since they produced neither programmed cell death nor metabolic stimulation.^167^

An interesting aspect of the SARs studies by Nicholson et al. is the proposed irreversible binding to the σ_2_R for the 6-isothiocyanate derivatives which is possibly responsible for their cytotoxic properties. This effect on cell viability has been examined by extensive washing of SK-N-SH neuroblastoma cells after an acute exposure with the tested compounds followed by an incubation period with fresh media. Particularly, the 6-isothiocyanate derivatives might mediate the irreversible binding via covalent bond formation with specific amino acid residues bearing a nucleophilic group (i.e., serine and cysteine) within the σ_2_R binding pocket. From our standpoint, an integrated approach involving the synthesis of a larger set of various properly substituted derivatives (e.g., Michael acceptors) and *in silico* molecular modeling studies might help to define the exact mechanism of the irreversible binding mode.

##### 3.2.3.3 6,7-Dimethoxy-1,2,3,4-tetrahydroisoquinoline Analogs

**Figure 25:**
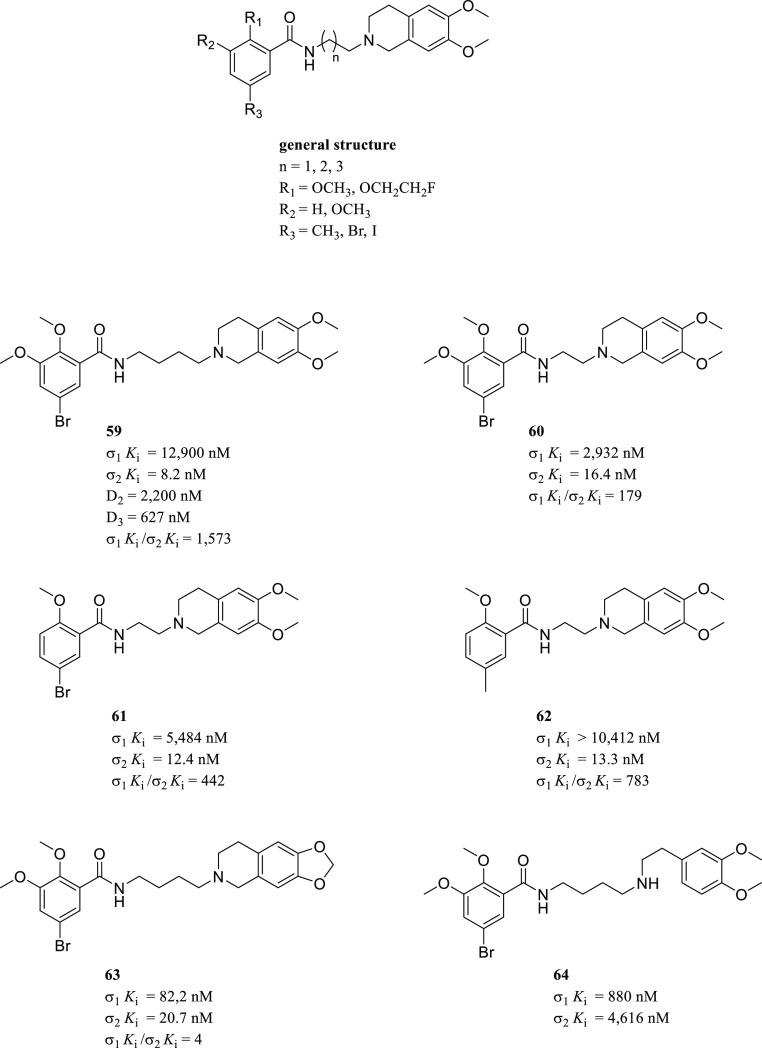
Chemical structure and σRs binding profile of early 6,7-dimethoxy-1,2,3,4-tetrahydroisoquinoline analogs **59**–**64**, reported by Mach and co-workers.

Similar to the *N*-cyclohexylpiperazine and the *N*-(4-fluorophenyl)piperazine, the 6,7-dimethoxy-1,2,3,4-tetrahydroisoquinoline moiety has been extensively used as a suitable σ_2_R-preferred cyclic amine fragment to develop selective σ_2_R ligands. To this extent, 6,7-dimethoxy-1,2,3,4-tetrahydroisoquinolinoalkyl benzamide derivatives (general structure, Figure 25) can be considered the most representative σ_2_R ligands prototype, even though Mach et al. initially developed them as a set of mixed dopamine receptor D_3_ and σ_2_R ligands.^168^ Indeed, since their discovery, this specific class of conformationally flexible amines showed high affinities and attractive selectivity for σ_2_R, making them useful chemical probes for imaging the σ_2_R in tumors with PET.^76,169^ A few examples of early developed 6,7-dimethoxy-1,2,3,4-tetrahydroisoquinoline analogs possessing a flexible benzamide scaffold (**59**–**64**) are depicted in Figure 25.^136,168^ SARs studies on this first set of ligands elucidated the structural features required for high σ_2_R affinity and selectivity. The introduction of the 6,7-dimethoxy-1,2,3,4-tetrahydroisoquinoline gave superb selectivity (σ_1_*K*_i_/σ_2_*K*_i_ = 1573) with a considerable reduction of binding with the dopamine receptors (**59**, Figure 25). Alkyl chain shortening, from four to two methylene units, did not affect the σ_2_R affinity (**59** vs **60**). Similarly, removing the methoxy group at the 3-position of the benzamide ring did not significantly reduce the σ_2_R affinity nor the selectivity (**61** vs **59** and **60**). The introduction of methyl instead of bromo group was highly tolerated (**62** vs **61**). Regarding the 1,2,3,4-tetrahydroisoquinoline moiety, fusing methylene-, ethylene-, and propylenedioxy rings onto the tetrahydroisoquinoline ring was detrimental for both the affinity and selectivity at the σ_2_R (**63** vs **59**). Furthermore, the tetrahydroisoquinoline ring-opening led to an ultimate loss of affinity for the σ_2_R (**64** vs **59**).^136,168^

**Figure 26:**
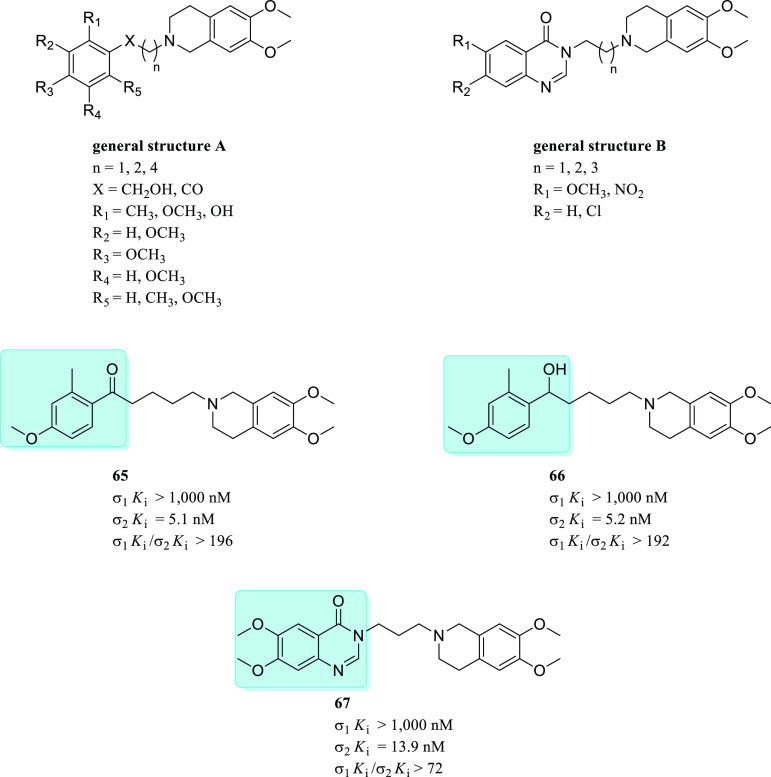
Chemical structure and σRs binding profile of 6,7-dimethoxy-1,2,3,4-tetrahydroisoquinoline analogs **65**–**67**.

As an extension of their previous works on selective σ_2_R ligands, Sun et al. synthesized a new series of 6,7-dimethoxy-1,2,3,4-tetrahydroisoquinoline analogs without the benzamide moiety.^170^ In this new series, substituted benzene and quinazolin-4(3*H*)-one fragments acting as electron-deficient or electron-rich aromatic portions were linked through different alkyl length chains to the 6,7-dimethoxy-1,2,3,4-tetrahydroisoquinoline moiety (general structures A and B, Figure 26). Unlike the quinazolin-4(3*H*)-one analogs, the new 6,7-dimethoxy-1,2,3,4-tetrahydroisoquinoline derivatives showed high σ_2_R affinities with a good selectivity ratio (**65** and **66**, Figure 26). Specifically, the ketone reduction to the corresponding hydroxyl group was broadly tolerated without affecting the affinity and selectivity at the σ_2_R (**65** vs **66**). On the other hand, the introduction of an electron-deficient aromatic moiety such as the quinazoline scaffold as a hydrophobic domain led to a decrease of affinity and selectivity (e.g., **67** vs **65**). Compound **66**, possessing an excellent selectivity ratio, produced a cytotoxicity effect toward two different cancer cell lines (EC_50_ = 12.50 μM for liver Huh-7, and EC_50_ = 14.86 μM for esophagus KYSE-140) similar to that of cisplatin (EC_50_ = 15.31 μM and 21.34 μM, respectively). Surprisingly, compound **65** which shows a close σRs binding profile to analog **66** did not show any effect, suggesting that the biological activity might not be σ_2_R mediated.

**Figure 27:**
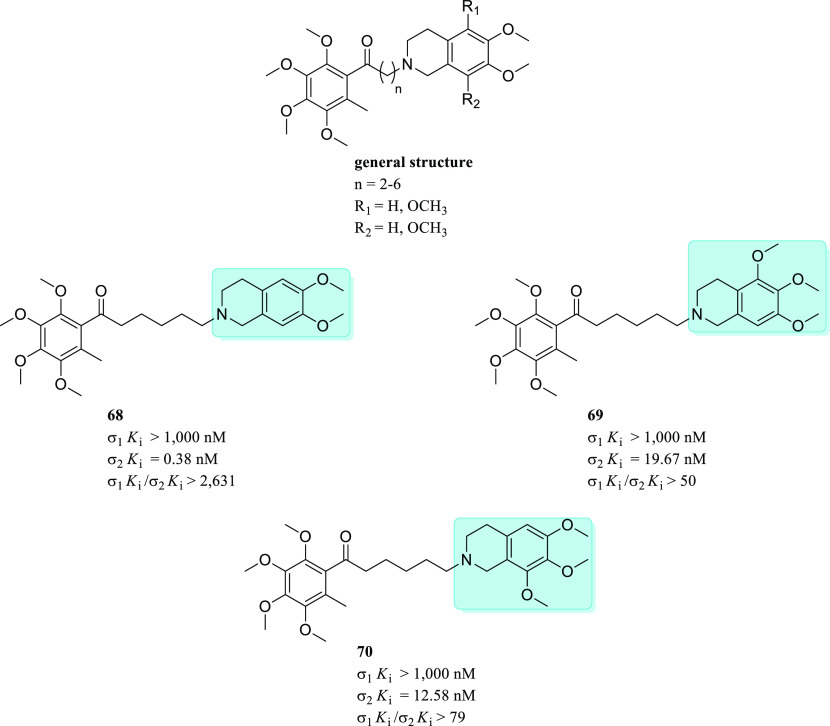
Chemical structure and σRs binding profile of 6,7-dimethoxy-1,2,3,4-tetrahydroisoquinoline analogs **68**–**70**.

Very recently, Xie et al. further developed the series mentioned above by analyzing the impact of introducing additional methoxy groups to the tetrahydroisoquinoline moiety on the σRs binding profile. In particular, to increase the affinity and selectivity toward the σ_2_R subtypes, the electron-rich 2,3,4,5-tetramethoxytoluene scaffold was used as a hydrophobic portion (general structure, Figure 27). The new di- and trimethoxy-substituted tetrahydroisoquinolin-2-alkylphenones showed moderate to high affinity and selectivity for the σ_2_R. Analog **68** (Figure 27), bearing a five methylene linker between the phenone carbonyl portion and the 6,7-dimethoxy-1,2,3,4-tetrahydroisoquinoline moiety, displayed the highest affinity and selectivity for the σ_2_R among all the benzamide derivatives reported so far (**68** vs **59** and **65**). Replacement of 6,7-dimethoxy-1,2,3,4-tetrahydroisoquinoline moiety with 5,6,7-trimethoxy- or 6,7,8-trimethoxy-1,2,3,4-tetrahydroisoquinoline moieties led to a decrease in affinity for σ_2_R (**68** vs **69** and **70**). Despite the favorable σRs binding profile, no significant inhibitory effects on MCF-7 cancer cell lines were observed. Indeed, functional studies performed by measuring intracellular calcium concentration allowed their classification as putative σ_2_R antagonists.^171^

**Figure 28:**
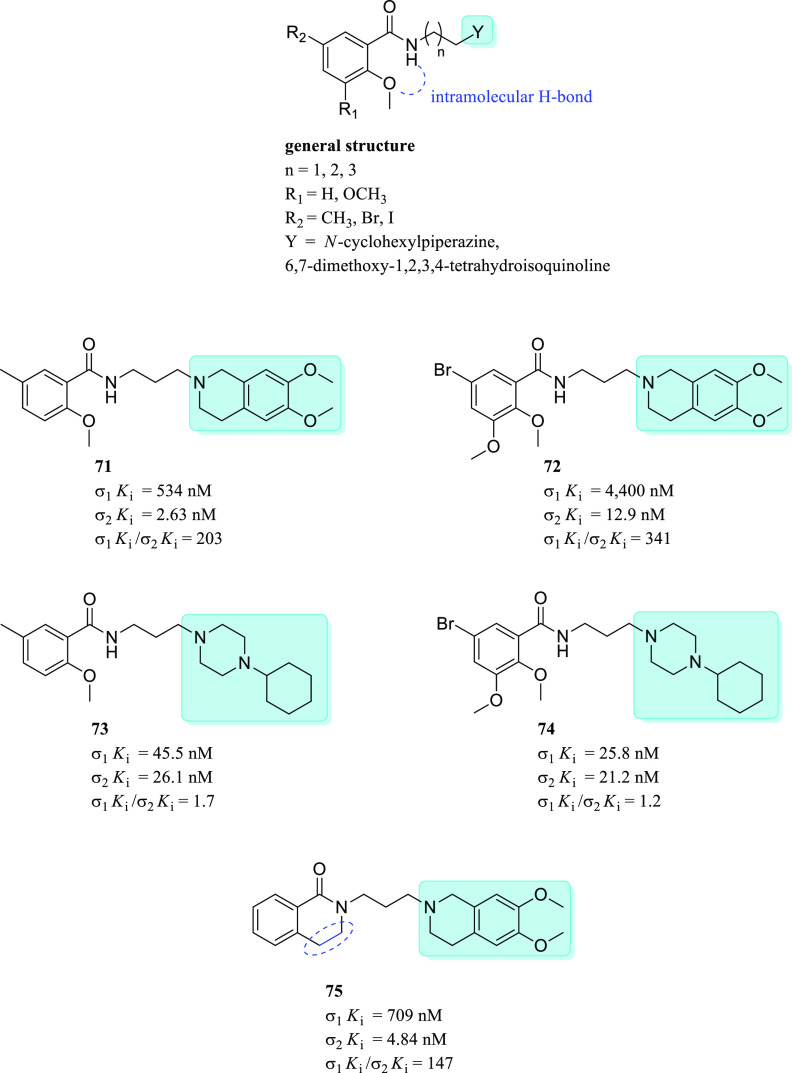
Chemical structure and σRs binding profile of compounds **71**–**75**.

In 2011, Abate et al. combined the structural determinants (i.e., benzamide scaffold and cyclic amine moieties) of their lead compound PB28 with the highly potent and selective σ_2_R ligands RHM-1 to develop new potential PET radiotracers.^172^ Good results in terms of σ_1_*K*_i_/σ_2_*K*_i_ selectivity ratio were obtained by 6,7-dimethoxy-1,2,3,4-tetrahydroisoquinoline derivatives **71** and **72** (Figure 28). However, the newly synthesized ligands also interacted with P-gp (e.g., EC_50_ = 2.5 μM for **72**), hence, limiting their further development as PET agents. A similar interaction with P-gp was observed for cyclohexylpiperazine analogs **73** and **74** (Figure 28), which also showed higher binding at the σ_1_R with a parallel loss of σ_2_R selectivity. These results are consistent with recent σ_1_R molecular models developed by Niso et al.^173^ which showed that the two methoxy substituents belonging to the tetrahydroisoquinoline ring might be placed in a sterically hindered region within the secondary hydrophobic domain of the σ_1_R binding pocket.

Interestingly, an intramolecular hydrogen bond between the 2-methoxy substituent and the *N*-atom of the benzamide group was proposed (general structure, Figure 28), suggesting a bicyclic-like active conformation for this set of derivatives.^172^ Indeed, this hypothesis was corroborated by the σRs binding profile of the 3,4-dihydroisoquinolin-1(2*H*)-one derivative **75** (Figure 28), in which the above-mentioned intramolecular bond has been mimicked by a rigid ring.^172^

**Figure 29:**
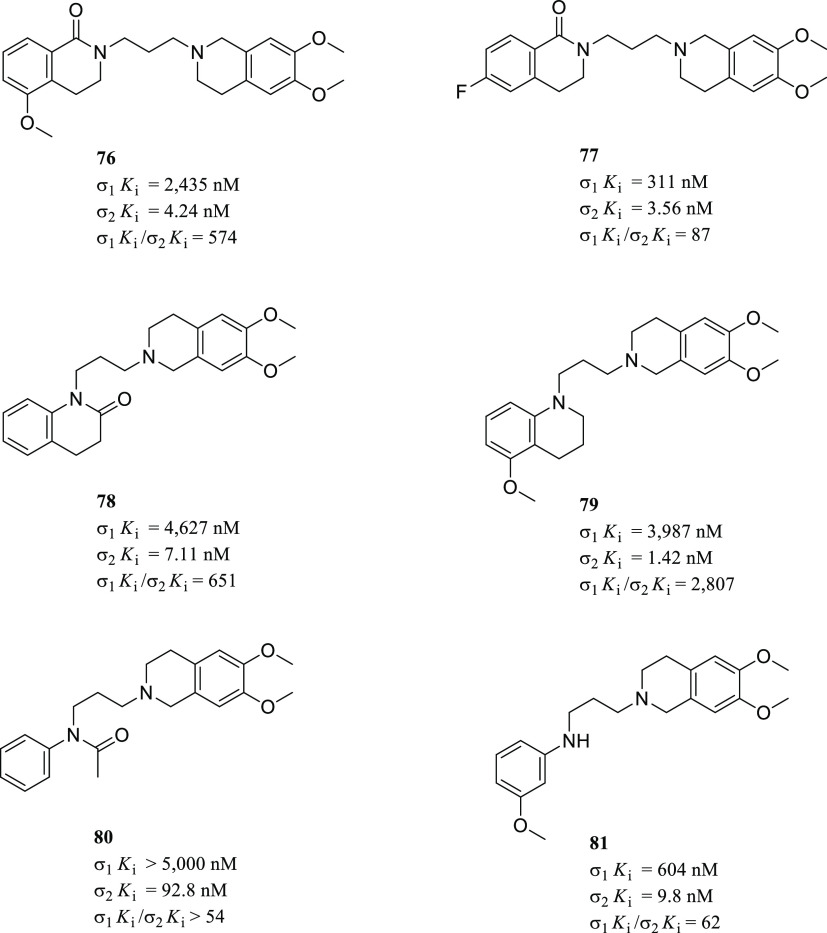
Chemical structure and σRs binding profile of compounds **76**–**81**.

Therefore, in a subsequent study, Niso et al. further investigated the role of bicyclic-preferred conformation proposed for flexible benzamides as a suitable hydrophobic portion to target the σ_2_R.^174^ The authors synthesized 3,4-dihydroquinolin-(1*H*)2-one and 1,2,3,4-tetrahydroquinoline derivatives along with flexible anilide and aniline analogs linked to the 6,7-dimethoxy-1,2,3,4-tetrahydroisoquinolinoalkyl portion. Also, considering the good σRs binding profile and the appropriate lipophilicity showed by previously developed substituted 3,4-dihydroisoquinolin(2*H*)1-one derivatives (**76** and **77**, Figure 29),^175^ the introduction of a 5-methoxy or 6-fluoro group in the new scaffolds was examined. Binding studies showed that 3,4-dihydroquinolin-(1*H*)2-one (**78**) and 1,2,3,4-tetrahydroquinoline (**79**) derivatives exhibited excellent affinity and selectivity for the σ_2_R, while the corresponding anilide (**80**) and aniline (**81**) analogs generally had a worse σRs binding profile (Figure 29). Notably, anilide derivatives showed a lower binding for the σ_2_R than the corresponding anilines, probably due to the lack of partial rigidification that might occur in anilines because of the lone pair conjugation of the *N*-atom with the benzene ring with a resulting resembling bicyclic framework.^174^ These data confirmed that a rigid bicyclic structure as a hydrophobic moiety was optimal for both affinity and selectivity for the σ_2_R. Surprisingly, none of the compounds exerted antiproliferative activity in human breast adenocarcinoma MCF-7 cells. However, since the modest interaction with the P-gp (EC_50_ = 2.13 μM), appropriate lipophilicity (clogP = 3.94), and the presence of easily radiolabeling functions (i.e., 3-methoxy groups) of **79**, the authors suggested its further development as a possible PET radiotracer.

##### 3.2.3.4 3-Alkoxyisoxazole Analogs

**Figure 30:**
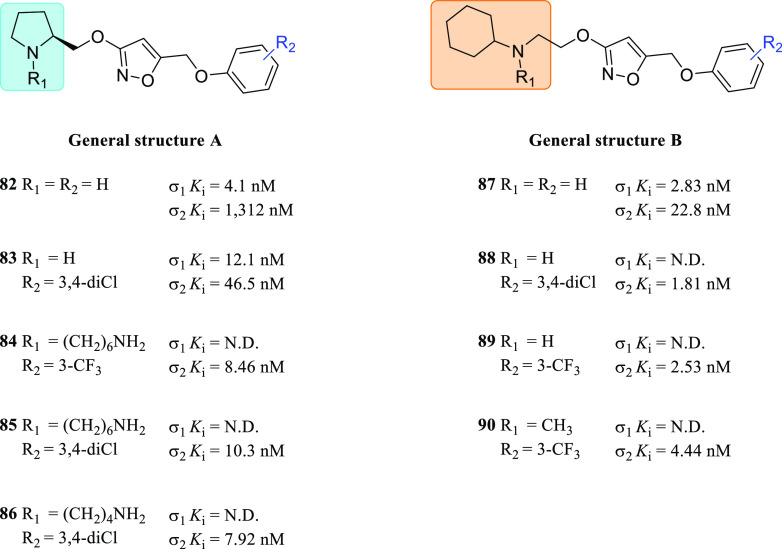
Chemical structures of 3-alkoxyisoxazoles **82**–**90** and their σRs binding profile.

Very recently, small molecules characterized by the presence of the 3-alkoxyisoxazole moiety have been designed and evaluated for their potential binding properties toward the σRs.^176^ This chemical scaffold was identified from the superimposition of the pharmacophoric elements required for a righteous binding to both σ_1_ and α4β2 nicotinic receptors.^177^ Compound **82** (Figure 30) was detected as a σ_1_R ligand with high affinity and selectivity over the σ_2_R subtype. Structural modifications of compound **82** were conducted to switch selectivity toward the σ_2_R and find potential anticancer compounds. The general structure of the developed 3-alkoxyisoxazole derivatives of **82** is depicted in Figure 30. Insertion of electron-withdrawing substituents (fluorine, chlorine, trifluoromethyl) on the aryloxymethyl group linked to the 5-position of the isoxazole ring led to increased affinity values for the σ_2_R subtype. Particularly, *meta*-fluorine and *meta*-trifluoromethyl substitutions were preferred concerning substitutions in *ortho* and *para* positions. The trifluoromethyl substitution was more effective with a 15-fold increased σ_2_*K*_i_ (data not shown) with respect to **82**. On the contrary, electron-donating substituents such as a methoxy group had minor effects on the affinity with only a 2-fold increased affinity for the σ_2_R. To further evaluate halogen substituents’ effects, double substitutions were performed, and optimal results were achieved with 3,4-dichloro-substituted aryl rings (compound **83**), with an increment of affinity of 28-fold compared to **82** (46.5 nM vs 1312 nM, respectively). N-methylation of the pyrrolidine ring of **82** slightly ameliorated the affinity for the σ_2_R, whereas replacement of the 2-pyrrolidine ring with its 3-pyrrolidine isomer led to a 2-fold improvement of σ_2_*K*_i_ values and a substantial reduction of σ_1_R affinity for **82** (data not shown). Retention of the electron-withdrawing substituents on the aromatic ring and insertion of a bulky aminoalkyl chain on the *N*-atom of 2-pyrrolidine gave compounds **84**–**86**, with an ameliorant of σ_2_*K*_i_ values from 4.5- to 6.0-fold when compared to compound **83**. Specifically, the best σ_2_*K*_i_ value for compounds with general structure A (Figure 30) was obtained when the alkyl chain was made of four carbon atoms (compound **86**, σ_2_*K*_i_ = 7.92 nM). Derivatives **87**–**90** (general structure B, Figure 30) were obtained by removing the pyrrolidine ring in favor of cycloalkylaminoethoxy moieties. Better results were obtained with unsubstituted six-membered rings. Indeed, the authors pointed out that smaller rings favor stronger interactions with the σ_1_R. On the other hand, more oversized rings increase the σ_2_ affinity and selectivity (compare **87** vs **82**). These results indicated that steric bulk plays an important role in the proper binding to the σ_2_R for this class of compounds. Starting from **87** (σ_2_*K*_i_ = 22.8 nM), favorable substitutions on the aromatic ring were repeated (i.e., insertion of electron-withdrawing substituents) in order to further validate the results previously discussed. As expected, insertion of a 3-trifluoromethyl group or a 3,4-dichloro substitution on **87** determined a strong increase of affinity for the σ_2_R getting one-digit values ranging from 1.81 nM (compound **88**) to 2.53 nM (compound **89**), while methylation of the nitrogen atom of the cycloalkylaminoethoxy group slightly reduced the affinity (compound **90**, 4.44 nM).

Compounds **82**–**90** were tested on two osteosarcoma cancer cell lines (143B and MOS-J cells). Despite their strong σ_2_R affinity, compounds **88**–**90** did not show significant cytotoxic properties as well as compounds **82**–**83** and **87**. Interestingly, the bulkier derivatives **84**–**86** displayed cytotoxicity in both cell lines, strengthening the previously discussed steric bulk hypothesis. In particular, in the crystal violet assay, compound **86** exhibited IC_50_ values of 0.89 μM and 0.71 μM for 143B and MOS-J cell lines. Compared with siramesine (IC_50_ = 1.81 μM and 2.01 μM for 143B and MOS-J), compound **86** possessed more potent cytotoxic properties. Cytotoxicity measured on healthy cells (human immortalized keratinocytes HaCaT and human normal embryonic liver cells LO2) revealed IC_50_ values of 6.47 μM and >10 μM, respectively. Compound **86** also caused inhibition of colony formation of osteosarcoma 143B cells and interference with the cell cycle reducing the number of cells in the S and G_2_M phases and blocking cells in the G_0_G_1_ phase. Cancer cell death induction was confirmed by the annexin V assay, where 33.4% of osteosarcoma cells started apoptosis when a concentration of 5 μM of **86** was applied. These results did not exclude an eventual involvement of σ_1_Rs in the cytotoxic properties of this class of compounds. Nevertheless, the 3-alkoxyisoxazole chemical scaffold could be further exploited to design novel σ_2_R ligands with augmented anticancer properties.

## 4 Conclusions

σRs represent a unique class of proteins involved in many physiopathological and pathological roles. Several immunohistochemical and radioligand binding assay studies revealed that both receptor subtypes are overexpressed in several cancer cell lines, suggesting a potential role of these proteins in cancer progression and tumor invasiveness. Moreover, the pharmacological modulation of σRs through small molecules has been proved to be a promising approach for developing novel therapeutics. However, although several studies supported the search for novel compounds targeting σRs to treat cancer, no compounds have reached the clinical phase yet. One of the reasons for this might be related to the heterogeneous and promiscuous biological effects exerted by certain σR ligands in preclinical studies, which are also related to the inconclusive evidence about the molecular role of the σRs in the etiopathogenesis and pathogenesis of cancer. Thus, there is still a need to answer crucial questions concerning the role and the involvement of σRs in tumor biology to reveal the real potential and benefit of the clinical use of σRs ligands in cancer chemotherapy. Indeed, an unambiguous characterization of the biological target is essential to link its perturbation to functional pharmacology. Moreover, several potent ligands, of both σRs subtypes, showed poor pharmacokinetic profiles. This fact hinders their clinical utilization as drugs and allows their use merely as diagnostic or pharmacological tools such as radioligand probes for PET scanning. We believe that this problem could be dealt with using the following strategies: (i) optimizing the ADME properties and the off-target effects, such as hERG binding, during the early stages of drug design; (ii) using computational techniques when available, such as QSAR and toxicity prediction machine learning methods, to estimate the potential adverse effects of the drug candidates; and (iii) repurposing of certain Food and Drug Administration approved CNS drugs which, by definition, have well-established pharmacokinetic and safety profiles. The drug repurposing approach is especially useful in the case of σRs ligands because they already share some pharmacophoric features with other ligands of certain CNS targets such as the opioid and dopamine receptors, which is exemplified by the affinity of pentazocine and haloperidol, respectively, to the σRs.

As extensively described in this perspective, tremendous efforts to discover selective σR ligands with antiproliferative properties have been made by medicinal chemists in the last 10 years. These efforts led to the identification of various chemical prototypes; therefore, for a comprehensive overview, a summary of this information has been collected in Table 3.

**Table 3:** Summary of Chemical Classification, Cancer Cell Lines, and Assays Used for the Biological Evaluation of the Most Representative σR Ligands

Compound	Chemotype	Cell line	Biological test	Reference
σ_1_R Ligands				
**1**	*N*,*N*-dialkyl and *N*-alkyl-*N*-aralkyl fenpropimorph-derivatives	NCI-H460, DU145, MCF7, SKOV-3, MB-MDA231	multiplex cytotoxicity assays	(101)
**2**		SKOV-3		
**5**		MCF-7		
**13**, **14**	spipethiane derivatives	MCF-7/ADR	annexin V-FITC assay, tail-flick assay	
**20**, **21**	spirocyclic thienopyran and thienofuran derivatives	A427	retinal ganglion assay, capsaicin assay, crystal violet assay, LDH assay	(83, 111, 113, 178)
**36**	7,9-diazabicyclo[4.2.2]decane derivative	A427	crystal violet assay	(131)
**ent-38**, **40**, **ent-40**, **ent-41**, **42**, **ent-42**	2,5-diazabicyclo[2.2.2]octane derivatives	A427	crystal violet assay, annexin V-FITC assay	(132)
**ent-38**, **39**, **ent-41**, **42**		5637		
σ_2_R Ligands				
**SWIII-123**	granatane derivative	SKOV-3, CaOV-3, BG-1	MTS assay	(146)
**49**	indole derivatives	MCF-7, MCF-7/dox, DU145, C6, A549/DX	MTT assay, cell cycle analysis	(158, 159, 162)
**51**		DU145, MCF-7, C6		(159)
**F281**	carbazole derivative	Panc02	MTT assay	(36)
**PB221**	tetralin derivative	Panc02, ALTS1C1, UN-KC6141	MTT assay, caspase-Glo assay	(36, 79)
**CM572**, **CM764**	1,3-benzoxazol-2(3*H*)-one derivatives	SK-N-SH	MTT assay	(112, 166)
**66**	6,7-dimethoxy-1,2,3,4-tetrahydroisoquinoline derivative	Huh-7, KYSE-140	CCK8 assay	(170)
**86**	3-alkoxyisoxazole derivative	143B, MOS-J	crystal violet assay, annexin V assay	(176)

It is worth noting that the σ_2_R identity has been established only recently, and no crystal structure has been reported yet. Nevertheless, a variety of new selective σ_2_R ligands have been recently discovered, and the specific overexpression of σ_2_Rs in a broad panel of cancer cell lines has been elucidated. As a result, the potential ability to pinpoint the tumor cells in an early stage of the pathology makes σ_2_R ligands powerful molecular tools exploitable in diagnostics and theranostics. Conversely, the study reported by Zeng et al.^37^ proved that the cytotoxicity exerted by some well-known σ_2_R ligands, including siramesine and PB28, was independent of the modulation of the σ_2_R. This finding corroborates the hypothesis that multiple unknown targets are likely involved in the observed cytotoxic effect mediated by σ_2_R ligands, making the overall scenario very intriguing. Besides, the recent identification by Abate and co-workers of σ_2_R ligands that promote collateral sensitivity in multidrug resistance cells further supports the hypothesis that a direct correlation between σ_2_Rs modulation and the observed cytotoxicity does not exist. Interestingly, collateral sensitivity is a well-studied phenomenon in cancer research.^179^ Thus, the development of new σR ligands which exploit the mechanism of the synthetic lethality to induce selective cytotoxicity is emerging as a new successful strategy in the field.^162^

Despite the lack of homogeneous evidence of one-to-one correspondence between σRs modulation and cytotoxicity, the involvement of these chaperons as key players in the tumor-supportive cellular machinery has been proved. Recently, Maher et al.^180^ described the ability of σRs ligands in regulating the programmed death-ligand 1 (PD-L1) expression and activity in cancer cells, suggesting a novel therapeutic strategy acting on tumor immune microenvironments. Specifically, σ_1_Rs inhibition either by σ_1_R negative modulators or using shRNA-induced PD-L1 autophagic degradation in breast and prostate cancer cells, suggesting the possibility of combining σRs modulators with specific drugs that can induce PD-L1 degradation (e.g., gefitinib), therefore enhancing the antitumor activity.^181^ However, the effectiveness of possible drug combinations might be mitigated by undesirable drug interactions and interferences. Alternatively, polypharmacology represents a current paradigm to enhance the efficacy of new anticancer agents.^182,183^ More specifically, in this case, selected molecular entities having the ability to intercept other validated molecular targets involved in the tumor progression and aggression can be effectively combined with structural determinants belonging to σR ligands to obtain novel multitarget ligands. As an example, Mangiatordi et al.^184^ have recently described their perspective on an innovative polypharmacology approach involving the concurrent targeting of cannabinoid receptor subtype 2 (CB_2_R) and σRs for cancer. Particularly, taking advantage of both the common pharmacophoric elements and the anticancer activities of CB_2_R agonists and σRs modulators, the authors proposed the development of molecular hybrids, that is, dual CB_2_R/σR ligands, potentially able to modulate different cancer pathways synergistically.

We expect the interest in the development of σRs ligands to continue for the next few years as tumor diagnostic tools as well as chemotherapeutic agents, perhaps as adjuvant therapies. Moreover, the availability of the σ_1_R crystal structure and the potential crystallization of the σ_2_R in the near future would give momentum to this research field. Finally, we believe that the recent discovery of repurposing some σRs ligands for fighting the early stages of COVID-19 could draw more attention to these biological targets. Altogether, this article represents a comprehensive literature review that might help to provide a reader with a perspective on the development of potent σRs ligands as additional weapons exploitable in anticancer therapy.

## Abbreviations Used

[^3^H]DTG: [^3^H]1,3-di(2-tolyl)guanidine
Bn: benzyl
CB_2_R: cannabinoid receptor subtype 2
Cl: clearance
CNS: central nervous system
CoMFA: comparative molecular field analysis
COVID-19: coronavirus disease 19
DOR: δ-opioid receptor
ent-: enantiomer
ER: endoplasmic reticulum
Et: ethyl
FITC: fluorescein isothiocyanate
GPCR: G protein-coupled receptor
HB: hydrogen bond
HBA: hydrogen-bond acceptor
HIF-1α: hypoxia-inducible factor 1α
HO-1: heme oxygenase-1
HY: hydrophobic
HYAl: hydrophobic aliphatic
HYAr: hydrophobic aromatic
*i*-Pr: isopropyl
KO: knockout
KOR: κ-opioid receptor
LDH: lactate dehydrogenase
LDL: low density lipoprotein
Me: methyl
MRP1: multidrug resistance-associated protein 1
MTS: 3-(4,5-dimethylthiazol-2-yl)-5-(3-carboxymethoxyphenyl)-2-(4-sulfophenyl)-2H-tetrazolium
MTT: 3-(4,5-dimethylthiazol-2-yl)-2,5-diphenyltetrazolium bromide
*n*-Bu: normal butyl
NMDA: *N*-methyl-d-aspartate
*n*-Pr: normal propyl
Nsp6: nonstructural protein 6
OXAIPN: oxaliplatin-induced peripheral neuropathy
PD-L1: programmed death-ligand 1
PET: positron emission tomography
PET–MRI: positron emission tomography–magnetic resonance imaging
P-gp: P-glycoprotein
PGRMC1: progesterone receptor membrane component 1
Ph: phenyl
PI: positive ionizable
*p*-Me: *para*-methyl
QSAR: quantitative structure–activity relationship
RNAi: RNA interference
ROS: reactive oxygen species
SAfiR: structure–affinity relationships
SAR: structure–activity relationships
SARS-CoV-2: severe acute respiratory syndrome coronavirus 2
SERT: serotonin transporter
shRNA: short hairpin RNA
*SIGMAR1*: sigma non-opioid intracellular receptor 1 gene
SMAC: second mitochondria-derived activator of caspase
TMEM97: transmembrane protein 97
σR: σ receptor
